# The DAF-16/FOXO Transcription Factor Functions as a Regulator of Epidermal Innate Immunity

**DOI:** 10.1371/journal.ppat.1003660

**Published:** 2013-10-17

**Authors:** Cheng-Gang Zou, Qiu Tu, Jie Niu, Xing-Lai Ji, Ke-Qin Zhang

**Affiliations:** Laboratory for Conservation and Utilization of Bio-Resources, Yunnan University, Kunming, Yunnan, China; Massachusetts General Hospital, Harvard Medical School, United States of America

## Abstract

The *Caenorhabditis elegans* DAF-16 transcription factor is critical for diverse biological processes, particularly longevity and stress resistance. Disruption of the DAF-2 signaling cascade promotes DAF-16 activation, and confers resistance to killing by pathogenic bacteria, such as *Pseudomonas aeruginosa*, *Staphylococcus aureus*, and *Enterococcus faecalis*. However, *daf-16* mutants exhibit similar sensitivity to these bacteria as wild-type animals, suggesting that DAF-16 is not normally activated by these bacterial pathogens. In this report, we demonstrate that DAF-16 can be directly activated by fungal infection and wounding in wild-type animals, which is independent of the DAF-2 pathway. Fungal infection and wounding initiate the Gαq signaling cascade, leading to Ca^2+^ release. Ca^2+^ mediates the activation of BLI-3, a dual-oxidase, resulting in the production of reactive oxygen species (ROS). ROS then activate DAF-16 through a Ste20-like kinase-1/CST-1. Our results indicate that DAF-16 in the epidermis is required for survival after fungal infection and wounding. Thus, the EGL-30-Ca^2+^-BLI-3-CST-1-DAF-16 signaling represents a previously unknown pathway to regulate epidermal damage response.

## Author Summary

In the natural environment, animals encounter different pathogens. Thus, different tissues within an organism must develop specific immune systems for survival. The epidermis acts as a physical barrier and represents a first line of defense against infection and physical injury in a variety of animals. Natural nematophagous fungi, such as *Drechmeria coniospora* and *Clonostachys rosea*, infect the epidermis of the roundworm *Caenorhabditis elegans* by producing conidia. Here we demonstrated that the DAF-16/FOXO transcription factor in the epidermis has a direct role in *C. elegans* defense against fungal infection and physical injury. We found that the EGL-30/EGL-8/IP3/ITR-1 signaling pathway triggers epidermal Ca^2+^ release through IP3 and its receptor ITR-1 after fungal infection. Ca^2+^ release induces the production of reactive oxygen species (ROS) by activating a dual-oxidase BLI-3. ROS in turn mediate DAF-16 activation in a Ste20-like kinase-1/CST-1-dependent manner. Thus, DAF-16 could act in a cell-autonomous way in the epidermis as an active regulator of immune responses to fungal infection and physical injury.

## Introduction

All organisms are in constant contacts with a variety of microorganisms. The innate immune system in hosts provides the first line of defense against these microorganisms. During the last decade, studies using *Caenorhabditis elegans* as a model host have revealed the involvement of evolutionarily conserved signaling pathways in the innate immune response to microbial infection and injury, including the DAF-2/DAF-16 insulin-like signaling pathway [1], [2]. *C. elegans* DAF-2 is orthologous to the mammalian insulin/insulin-like growth factor-1 receptor [3] and *daf-2* mutants exhibit increased resistance to pathogenic bacteria, such as *Pseudomonas aeruginosa* and *Staphylococcus aureus* [4]. Under standard growth conditions, DAF-2 initiates a kinase cascade that leads to the phosphorylation and cytoplasmic retention of its downstream effector DAF-16, the ortholog of mammalian Forkhead box O (FOXO) transcription factors 5,6,7. A reduction in DAF-2 signaling leads to the dephosphorylation of DAF-16, allowing its nuclear translocation and transcriptional activation [5], [6]. The pathogen-resistant phenotype of *daf-2* mutants is suppressed by mutations in *daf-16*, suggesting a crucial role for DAF-16 in innate immunity against bacteria [4]. As a transcriptional factor, activated DAF-16 mediates a variety of genes that are positive regulators of innate immunity against pathogenic bacteria [8], [9]. In *Drosophila* and human tissues, FOXOs also induce the expression of a variety of antimicrobial peptides, such as drosomycin and defensins [10], suggesting that the role for FOXOs as innate immunity regulators is highly conserved across species.

Although DAF-16 is involved in immune responses to pathogenic bacteria including *P. aeruginosa*, *S. aureus*, *Enterococcus faecalis* and *Salmonella enterica*, *daf-16* mutants are not significantly more susceptible than wild-type worms to the killing mediated by these bacteria [4], [11]. Interestingly, a previous study shows that although the knock-down of *daf-16* by RNAi in wild-type worms does not affect susceptibility to *P. aeruginosa* PA14, intestinal-specific knock-down of *daf-16* leads to enhanced susceptibility to PA14 [7]. These results suggest that DAF-16 in the intestine, but not in the whole worms, is required for resistance to PA14 infection. One reasonable explanation is that loss of DAF-16 in the intestine, in combination with loss of DAF-16 in other tissues, has an overall neutral effect on resistance to PA14 infection. Meanwhile, two recent studies demonstrate that two bacterial pathogens enteropathogenic *Escherichia coli* (EPEC) and *Bacillus thuringiensis* induce DAF-16 nuclear translocation, respectively [12],[13]. These results contradict the previous notion that DAF-16 is activated by something other than pathogens [11], [14]. More importantly, *daf-16* mutants are more sensitive to the two bacterial pathogens [12], [15], [16]. However, the mechanism underlying DAF-16 activation by these bacterial pathogens remains unclear.

Pathogenic bacteria, including *P. aeruginosa*, *S. aureus*, *E. faecalis*, *S. enterica*, and human pathogenic yeast *Candida albicans* infect the nematode intestine [2], [17], whereas natural nematophagous fungi, such as *Drechmeria coniospora* and *Clonostachys rosea*, infect the epidermis of nematode, leading to epidermal cell damage [18], [19], [20], [21], [22]. When comparing gene expression profiles of *C. elegans* infected with *D. coniospora* [23] and predicted DAF-16 transcriptional target genes [8], we found that there was a significant overlap between *D. coniospora*-upregulated genes and DAF-16 target genes. These findings prompted us to examine the role of DAF-16 in the innate immune response to fungal infection. After exposure of *C. elegans* to *D. coniospora* and *C. rosea*, we found that *daf-16* mutants were more susceptible than wild-type worms to killing by fungi. Further studies indicated that fungal infections resulted in the activation of DAF-16 as a consequence of the production of reactive oxygen species (ROS). Similar results were obtained with nematodes subjected to physical injury. Our data demonstrate that DAF-16 can act in a tissue-specific way in the epidermis as an active regulator of immune responses to fungal infection and physical injury.

## Results

### Fungal infection and physical wounding activate DAF-16 independently of the insulin/IGF-1 pathway

**Figure 1:**
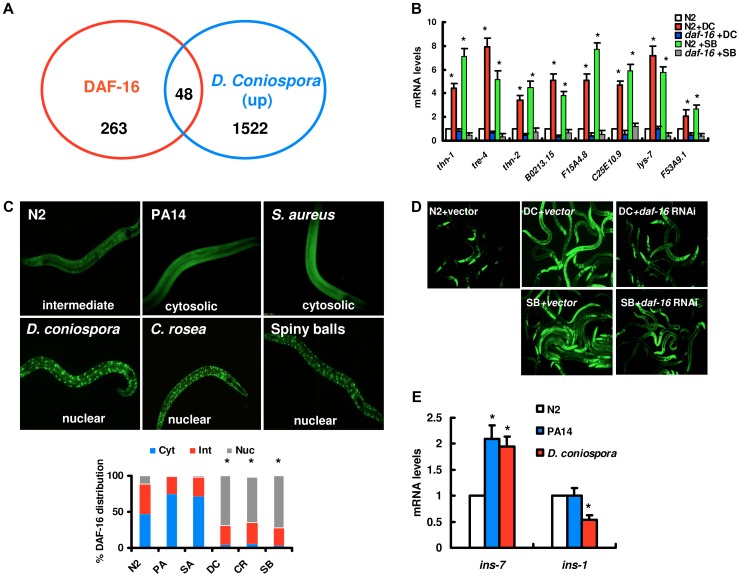
DAF-16 is activated by fungal infection and physical injury. (A) Venn diagrams comparing the overlaps in genes activated by *D. coniospora* and the target genes of DAF-16. (B) qPCR analysis of expression of DAF-16 target genes in wild-type (N2), and *daf-16(mu861)* mutants 24 h after *D. coniospora* (DC) infection or treatment with spiny balls. *P<0.05, N2+DC or N2+SB relative to N2. (C) DAF-16 translocation assay. Transgenic worms expressing DAF-16::GFP were treated with *P. aeruginosa* PA14 (PA), *S. aureus* (SA), *D. coniospora* (DC) and *C. rosea* (CR), and spiny balls (SB). After 12 h of treatment, the DAF-16::GFP expression pattern was observed. DAF-16 is present in cytosolic (Cyt), intermediate (Int), or nuclear (Nuc) fractions. Quantification of DAF-16 distribution. These results are means±SD of four experiments. *P<0.05 *versus* control (N2). n = 100–110 nematodes per condition. (D) Expression of *Psod::GFP* was up-regulated in WT animals exposed to *D. coniospora* or spiny balls for 12 h. *daf-16* RNAi inhibited the expression of *Psod-3::GFP* induced by *D. coniospora* or spiny balls. (E) The mRNA levels of DAF-2 insulin-like signaling ligands, *ins-7* and *ins-1*, in wild-type animals exposed to *P. aeruginosa* PA14 and *D. coniospora* for 12 h, respectively. These results are means±SD of four experiments. * *P<*0.05 *versus* control (N2).

Under standard growth conditions, DAF-16 is distributed predominately throughout the cytoplasm of all tissues [5], [6], [7]. We compared previously identified DAF-16 target genes [8] to published microarray analysis of gene expression in response to *D. coniospora* infection [23]. 48 of the genes up-regulated by *D. coniospora* are also targets of DAF-16 (Figure 1A, Table S1), significantly more than expected by chance (Fisher's exact test, *P*<0.0001). To further confirm these results, we randomly selected eight of these genes and determined their expression by qPCR (Figure 1B). The expression of these eight genes was significantly elevated after *D. coniospora* infection. However, *daf-16* mutation suppressed the up-regulation of these eight genes induced by *D. coniospora*. These results suggest that nematophagous fungi could activate the transcription activity of DAF-16 in wild-type worms under standard growth conditions.

To test this hypothesis, we monitored the cellular translocation of DAF-16 using transgenic worms that express a functional DAF-16::GFP fusion protein. The status of DAF-16 localization was categorized as cytosolic, intermediate, or nuclear (Figure 1C). We observed that exposure to *D. coniospora* or *C. rosea* induced DAF-16 nuclear localization (Figure 1C). In contrast, infection with *P. aeruginosa* PA14 or *S. aureus* ATCC 25923 failed to cause increased DAF-16 nuclear accumulation (Figure 1C), consistent with previous studies [7]. Recent studies have demonstrated that fungal infection and epidermal injury activate similar signaling pathways in *C. elegans* 19,24. Infection by nematophagous fungi causes nematode cuticle damage [18], [19], [20], [21], [22]. We have previously reported a unique fungal structure, called the spiny ball, on the vegetative hyphae of the fungus *Coprinus comatus* that damages the nematode cuticle [25]. To investigate the response to physical wounding of the cuticle, we exposed worms to purified *C. comatus* spiny balls. After nematodes were added to NGM plates containing purified spiny balls (approximately 10,000/plate), DAF-16 nuclear localization was observed (Figure 1C). Meanwhile, the expression of the eight genes was significantly up-regulated in wild-type worms, but not in *daf-16(mu86)* mutants, after treatment with spiny balls (Figure 1B). We also tested one of classic targets of DAF-16, *sod-3*, using transgenic worms that express *Psod-3::GFP*. We found that infection of *D. coniospora* or treatment with spiny balls up-regulated the expression of *Psod-3::GFP* (Figure 1D). Knock-down of *daf-16* by RNAi inhibited the expression of *Psod-3::GFP* induced by *D. coniospora* or spiny balls. It should be noted that, similar to fungal infection (Figure S1A and S1B in Text S1), mutation in *daf-2(e1370)* also induced DAF-16 nuclear translocation predominately both in the hypodermis and the intestine without fungal infection (Figure S1C in Text S1). Meanwhile, we found that either epidermal- or intestinal-specific knock-down of *daf-16* by RNAi suppressed the expression of the eight DAF-16 target genes (Figure S2A and S2B in Text S1). Taken together, these results demonstrate that infection by nematophagous fungi and physical wounding activate DAF-16 in *C. elegans*.

Reduced signaling in the DAF-2 pathway results in the nuclear accumulation of DAF-16 [5], [6]. *P. aeruginosa* PA14 infection up-regulates the expression of the insulin-like agonist *ins-7*, thus activating the DAF-2 insulin-like signaling [7], [26]. This is one of the mechanisms by which PA14 suppresses nuclear accumulation of DAF-16. It is tempting to speculate that in contrast to bacterial infection, fungal infection reduces expression of insulin-like agonists, thereby leading to the activation of DAF-16. Unexpectedly, like *P. aeruginosa* PA14, *D. coniospora* also up-regulated the expression of *ins-7* (Figure 1E). We thus examined the effect of *ins-7* on DAF-16 translocation and the immune phenotypes, and found that mutations in *ins-7* did not alter DAF-16 translocation and the survival of worms after *D. coniospora* infection and treatment with spiny balls (Figure S3A–C in Text S1). In addition, the expression of *ins-1*, an antagonist of DAF-2 signaling [27], [28], was down-regulated after *D. coniospora* infection (Figure 1E). These results suggest that similarly to bacterial infection, fungal infection also activates DAF-2 insulin-like signaling, probably by altering the expression of insulin-like peptides. Thus, the activation of DAF-16 does not result from reduced signaling in the DAF-2 pathway, suggesting that other mechanisms exist for the activation of DAF-16 following fungal infection.

### DAF-16 in the epidermis is required for the immune response to fungal infection and physical injury

**Figure 2:**
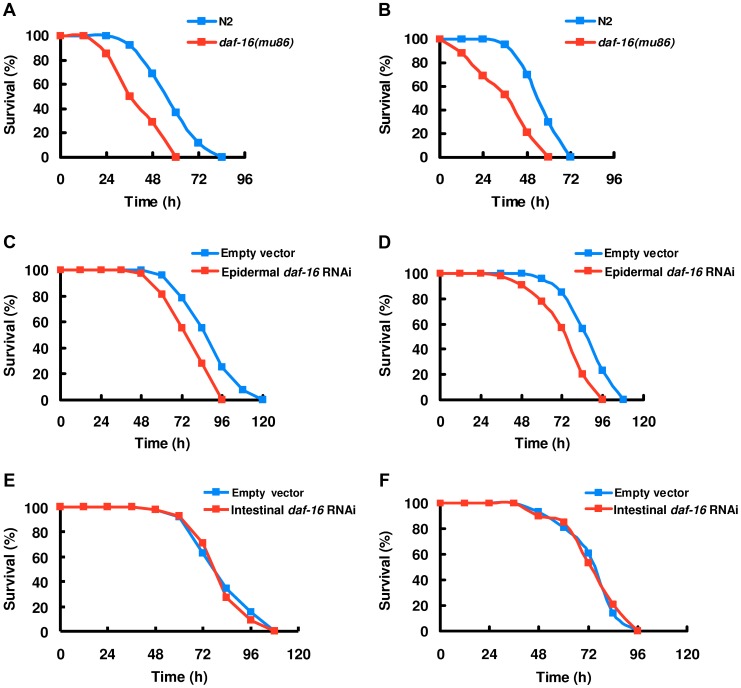
DAF-16 in the epidermis is required for resistance to fungal infection and physical injury. (A–B) *daf-16* mutants were sensitive to fungal infection and spiny balls. Fraction of *daf-16(mu86)* and wild-type animals are plotted as a function of time exposure to *D. coniospora* (A), and spiny balls (B). *P*<0.001 relative to wild-type animals. (C–D) Contribution of epidermal DAF-16 to sensitivity by fungi and injury. Epidermal-specific RNAi of *daf-16* significantly reduces survival rate of worms exposed to *D. coniospora* (C), and spiny balls (D). *P*<0.01 relative to control with empty vector (NR222). (E–F) Intestinal-specific *daf-16* RNAi had no effect on sensitivity to *D. coniospora* infection (E), and physical injury (F).

Since fungal infection activated DAF-16, we determined the survival rates of *daf-16(mu86)* mutants after infection by *D. coniospora* and *C. rosea*. We found that *daf-16(mu86)* mutants exhibited enhanced susceptibility to killing by *D. coniospora* (Figure 2A) and *C. rosea* (Figure S4A in Text S1). Similar results were obtained from worms by *daf-16* RNAi (Figure S4B and S4C in Text S1). These results indicate that DAF-16 is directly involved in controlling fungal resistance in wild-type animals. Meanwhile, we also examined the survival of worms in the presence of spiny balls. *daf-16(mu86)* animals were more sensitive than wild-type animals to physical injury (Figure 2B).

Unlike pathogenic bacteria that mainly infect the intestine, nematophagous fungi infect the epidermis [23]. To determine tissue-specific activities of DAF-16 in the regulation of immune responses to fungal infection and physical injury, we knocked down *daf-16* by RNAi in the intestine, the epidermis, and muscle, respectively. We found that epidermal-specific knock-down of *daf-16* resulted in enhanced sensitivity to *D. coniospora* infection (Figure 2C) and physical injury by spiny balls (Figure 2D). The epidermal-specific knock-down of *daf-16* did not alter DAF-16 nuclear translocation in the intestine after *D. coniospora* infection (Figure S5A and S5B in Text S1). In contrast, intestinal- or muscular-specific *daf-16* RNAi had no effect on sensitivity to *D. coniospora* infection (Figure 2E, Figure S6A in Text S1) and spiny balls (Figure 2F, Figure S6B in Text S1). In addition, expression of *daf-16* under control of an epidermal (*dpy-7*) promoter [29] enhanced the resistance to *D. coniospora* infection and physical injury in wild-type animals (Figure S7A and S7B in Text S1). We conclude that DAF-16 functions within the epidermis of nematode to promote immune responses to fungal infection and physical wounding.

### ROS production is induced through BLI-3 during fungal infection and physical wounding

**Figure 3:**
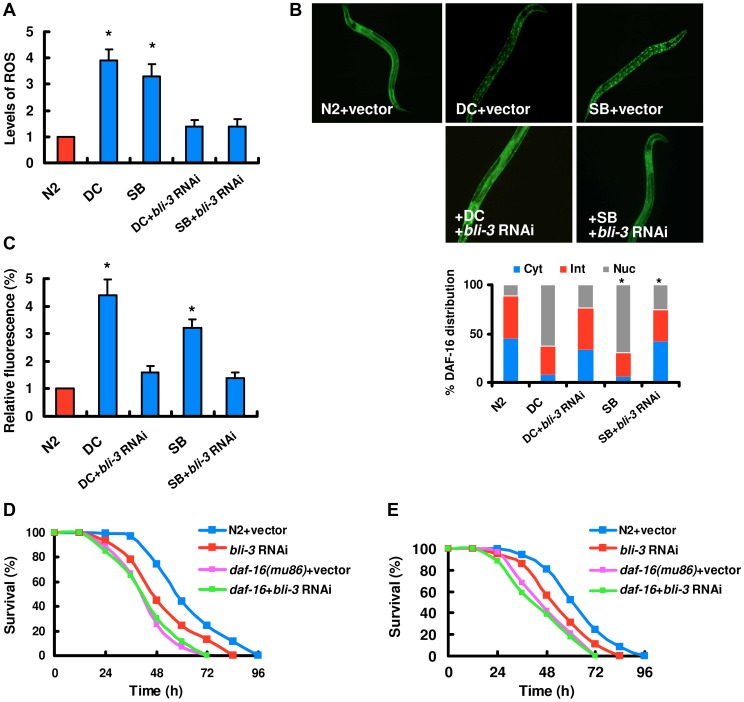
BLI-3-mediated ROS formation plays a crucial role in DAF-16 nuclear accumulation. (A) The levels of ROS were elevated in wild-type worms after fungal infection and treatment with spiny balls. Worms exposed to *D. coniospora* (DC), and spiny balls (SB) for 8 h. The levels of ROS were detected by DCF-DA. The induction of ROS by *D. coniospora* and spiny balls were abolished by knock-down of *bli-3* RNAi. The data are expressed as percent of control (N2). These results are means±SD of four experiments. *P<0.05 *versus* control (N2). (B) DAF-16 nuclear translocation was diminished by *bli-3* RNAi. Transgenic worms expressing DAF-16::GFP subjected to *bli-3* RNAi were treated with *D. coniospora* (DC) and spiny balls (SB). After 12 h of treatment, the DAF-16::GFP expression pattern was observed. The lower part shows quantification of DAF-16 distribution. n = 100–110 nematodes per condition. These results are means±SD of four experiments. *P<0.05 *versus* control (DC and SB). (C) Expression of *Psod::GFP* was up-regulated in WT animals exposed to *D. coniospora* or spiny balls for 12 h. *bli-3* RNAi inhibited the expression of *Psod-3::GFP* induced by *D. coniospora*. *P<0.05 *versus* control (N2). (D and E) Contribution of BLI-3 to fungal infection and physical injury sensitivity. *bli-3* RNAi significantly reduced the survival rate of wild-type worms exposed to *D. coniospora* (D) and spiny balls (E). *P*<0.001 relative to control with empty vector. However, *bli-3* RNAi did not enhanced susceptibility of *daf-16(mu86)* mutants to killing by *D. coniospora* (D) and spiny balls (E).

Accumulating evidence suggests that the levels of ROS in tissues are induced in response to physical wounding in human epithelial keratinocytes [30], [31], [32], the tail fin of zebrafish larvae [30], [31], [32], and the Drosophila embryo epidermis [30], [31], [32]. Since oxidative stress induces the activation of DAF-16 in *C. elegans* [33], we hypothesized that the production of ROS is one of the mechanisms underlying DAF-16 activation by fungal infection and physical injury. To test this idea, we first determined the levels of ROS using 2′,7′-dichlorodihydrofluorescein diacetate (H_2_DCFDA), a fluorescent dye that has been used to detect the ROS levels in *C. elegans* [34], [35], [36]. We found that the levels of ROS were dramatically elevated during fungal infection and treatment with spiny balls (Figure 3A).

Recent studies demonstrate that dual oxidases (DUOXs) mediate ROS production during wound responses in zebra fish larvae and Drosophila embryos [31], [32]. In *C. elegans*, there are two DUOX homologs. BLI-3/Ce-DUOX-1 is the major enzyme responsible for the production of ROS [37]. Since mutations in *bli-3* and the standard feeding RNAi with construct based on *bli-3* result in a severe blistered phenotype [38], [39], we tested the worms subjected to RNAi in a 1/10 dilution as described by Chavez et al. [38], [39]. qPCR analysis demonstrated that that knock-down of *bli-3* in a 1/10 dilution reduced more than 50% *bli-3* mRNA levels (Figure S8 in Text S1). We found that the knock-down of *bli-3* by RNAi significantly reduced the ROS levels induced by *D. coniospora* and spiny balls (Figure 3A), indicating that BLI-3 is involved in the increase in ROS levels in these processes. Furthermore, the nuclear accumulation of DAF-16 was markedly reduced by *bli-3* RNAi after infection of *D. coniospora* and treatment with spiny balls, respectively (Figure 3B). Similarly, knock-down of *bli-3* by RNAi markedly inhibited the expression of *Psod-3::GFP* induced by *D. coniospora* and spiny balls (Figure 3C). Taken together, these results suggest that ROS production is essential for the activation of DAF-16 after fungal infection and physical injury.

Meanwhile, *bli-3* RNAi enhanced susceptibility to killing by *D. coniospora* and spiny balls (Figure 3D and 3E). However, in *daf-16(mu86)* background, knock-down of *bli-3* by RNAi did not cause an increase in susceptibility to *D. coniospora* and spiny balls compared to *daf-16(mu86)* mutants alone. *bli-3* is mainly expressed in the epidermis of nematodes [38]. We thus used tissue-specific RNAi to reduce *bli-3* function only in the adult epidermis. As expected, after *D. coniospora* infection and treatment with spiny balls, epidermal-specific RNAi of *bli-3* significantly suppressed the production of ROS (Figure S9A in Text S1), inhibited nuclear accumulation of DAF-16 (Figure S9B in Text S1), and reduced survival rate of worms (Figure S9C and S9D in Text S1). In contrast, intestinal-specific knock-down of *bli-3* had no such effects (Figure S9E and S9F in Text S1). These results suggest that BLI-3 functions within the epidermis to promote ROS formation in response to fungal infection and physical injury.

BLI-3 is a dual oxidase, which has a NADH oxidase activity and a peroxidase activity [38]. The mutant *bli-3(e767)* encodes a protein that lacks the peroxidase domain, but retains its ability to produce ROS. *bli-3(e767)* mutants exhibited similar sensitivity to killing by *D. coniospora* and spiny balls as did wild-type animals (Figure S10A and S10B in Text S1), indicating that the peroxidase activity of BLI-3 is not crucial for resistance to fungal infection and physical injury.

### The IP3-ITR-1/Ca^2+^ signaling functions upstream of BLI-3 to regulate DAF-16 nuclear accumulation

**Figure 4:**
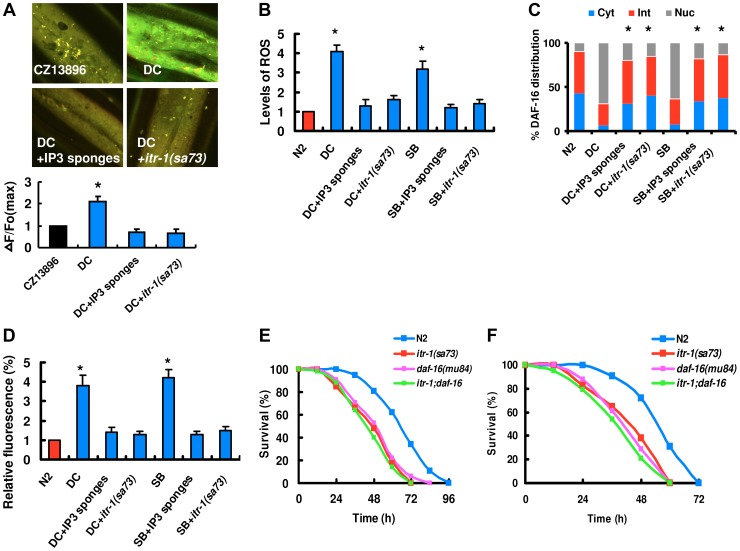
The IP3-ITR-1-Ca^2+^ pathway regulates DAF-16 nuclear accumulation. (A) Epidermal GCaMP was induced after fungal infection. IP3 sponges overexpression and mutations in *itr-1* inhibited GCaMP fluorescence after *D. coniospora* infection (DC), respectively. These results are means±SD of four experiments. The right part shows quantification of GCaMP fluorescence levels. The data are expressed as percent of the strain CZ13896. *P<0.05 *versus* control (CZ13896). (B) IP3 sponges overexpression and mutations in *itr-1* suppressed the levels of ROS after *D. coniospora* infection. *P<0.01 *versus* control (N2). (C) Quantification of DAF-16 distribution. n = 100–110 nematodes per condition. These results are means±SD of four experiments. *P<0.05 *versus* control (DC and SB). (D) IP3 sponges overexpression and mutations in *itr-1* inhibited the expression of *Psod-3::GFP* induced by *D. coniospora* infection and spiny balls for 12 h. *P<0.05 *versus* control (N2). (E–F) *itr-1(sa73)* mutants exhibited increased susceptibility after infection of *D. coniospora* (E) and treatment with spiny balls (F). *P*<0.001 relative to control with empty vector. However, mutations in *itr-1* did not enhanced susceptibility of *daf-16(mu86)* mutants to killing by *D. coniospora* (E) and spiny balls (F).

How does fungal infection activate DUOX1? BLI-3 contains a Ca^2+^-responsive EF hand domain [38], implicating that Ca^2+^ probably plays a role in regulating the activity of BLI-3 for ROS production in response to fungal infection. We thus determined Ca^2+^ release using the nematode strain carrying Ca^2+^ sensor GCaMP3 under the control of epidermal-specific promoters. As shown in Figure 4A, *D. coniospora* infection induced an increase in GCaMP fluorescence. These results indicate that fungal infection induces Ca^2+^ release in the epidermis. Increases in intracellular Ca^2+^ are initiated by the phospholipase C (PLC) family of enzymes, which hydrolyze phosphatidylinositol 4,5-diphosphate (PIP2) to produce inositol 1,4,5-trisphosphate (IP3) and diacylglycerol [40]. Since IP3 and its receptor IP3R/ITR-1 contribute to the epidermal Ca^2+^ release after needle wounding [29], we tested the role of the IP3/ITR-1 signaling in Ca^2+^ release after *D. coniospora* infection. We used worms overexpressing N-terminal IP3 binding domains (“IP3 sponges” (cz12690)) in the epidermis [29]. IP3 sponges function as a dominant negative regulator to disturb IP3 signaling. We observed that IP3 sponges led to a decrease in GCaMP fluorescence. GCaMP fluorescence was reduced in *itr-1(sa73)* mutants (Figure 4A). Thus, abolishment of the IP3/ITR-1 signaling cascade inhibited Ca^2+^ release after fungal infection.

Next, we tested whether Ca^2+^ release is required for the formation of ROS and DAF-16 nuclear accumulation after fungal infection and physical injury. An increase in the production of ROS and DAF-16 nuclear accumulation was essentially abolished in worms expressing IP3 sponges in the epidermis after fungal infection and physical injury (Figure 4B and 4C). Meanwhile, blockage of Ca^2+^ release by mutations in *itr-1* also suppressed the production of ROS and DAF-16 nuclear accumulation after *D. coniospora* infection and treatment with spiny balls (Figure 4B and 4C). Finally, we found that overexpression of IP3 sponges or knock-down of *itr-1* by RNAi markedly inhibited the expression of *Psod-3::GFP* induced by *Drechmeria coniospora* and spiny balls (Figure 4D). These results demonstrate that the IP3/ITR-1/Ca^2+^ signaling cascade is genetically upstream of BLI-3 for DAF-16 activation.

We asked whether blockage of Ca^2+^ signaling could influence the survival rate after fungal infection and physical injury. Indeed, worms expressing IP3 sponges in the epidermis were more susceptible than wild-type worms to killing mediated by *D. coniospora* and spiny balls, respectively (Figure S11A and S11B in Text S1). Furthermore, mutations in *itr-1(sa73)* shifted the survival curve to mimic the *daf-16(mu86)* phenotype after *D. coniospora* infection (Figure 4E) and treatment with spiny balls (Figure 4F). However, the survival curve for *daf-16(mu86); itr-1(sa73)* double mutants was similar to that of *daf-16(mu86)* mutants. In *C. elegans*, *itr-1* is expressed in many tissues, including the epidermis [41]. We found that epidermal-specific RNAi of *itr-1* significantly reduced the survival of worms after infection of *D. coniospora* and treatment with spiny ball (Figure S12A and S12B in Text S1). In contrast, intestinal-specific knock-down of *itr-1* had no such effects (Figure S12C and S12D in Text S1). These results indicate that the IP3/ITR-1 pathway functions within the epidermis to promote innate immunity against fungal challenge and physical injury.

### EGL-30 and EGL-8 regulate DAF-16 nuclear accumulation

**Figure 5:**
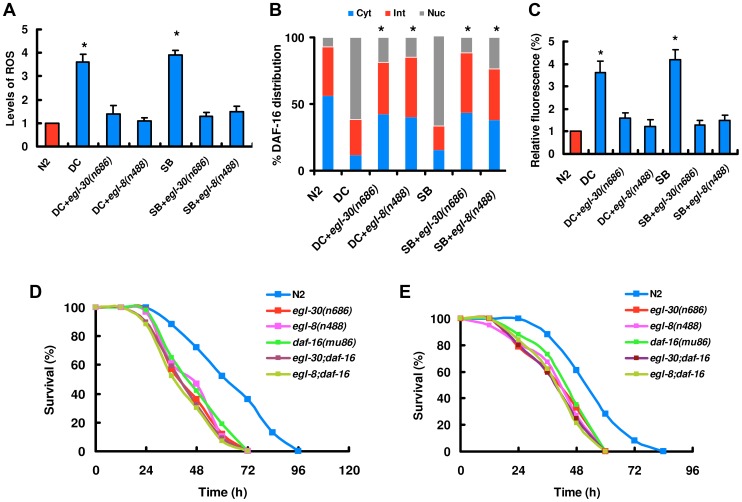
The Gαq-PLCβ signaling is required for DAF-16 nuclear accumulation. (A) *egl-30(n686)* or *egl-8(n488)* mutants displayed reduced the production of ROS induced by *D. coniospora* infection (DC) and spiny balls (SB). These results are means±SD of four experiments. * *P<*0.05 *versus* control (N2). (B) Quantification of DAF-16 distribution. n = 100–110 nematodes per condition. These results are means±SD of four experiments. *P<0.05 *versus* control (DC and SB). (C) Mutations in *egl-30 and egl-8* inhibited the expression of *Psod-3::GFP* induced by *D. coniospora* infection and treatment with spiny balls or 12 h. *P<0.05 *versus* control (N2). (D–E) Mutations in *elg-30* and *egl-8* reduced the survival rate of worms exposed to *D. coniospora* (D) and spiny balls (E). *P*<0.001 relative to control with wild-type worms. However, mutations in *egl-30* or *egl-8* did not enhanced susceptibility of *daf-16(mu86)* mutants to killing by *D. coniospora* (D) and spiny balls (E).

Since needle wounding in *C. elegans* triggers an EGL-30-EGL-8 signaling cascade, leading to the release of Ca^2+^ [29], we tested whether the Gαq protein EGL-30 and the phospholipase C (PLCβ) EGL-8 were also required for the production of ROS and DAF-16 nuclear accumulation upon fungal infection and physical injury. We found that the formation of ROS was reduced in *egl-30(n686)* or *egl-8(n488)* mutants after infection of *D. coniospora* and treatment with spiny balls (Figure 5A). To confirm the role of *egl-30* and *egl-8* in the activation of DAF-16, we crossed the *egl-30* or *egl-8* mutations into the transgenic worms that express DAF-16::GFP fusion protein. As shown in Figure 5B, the nuclear accumulation of DAF-16::GFP was reduced in *egl-30(n686)* or *egl-8(n488)* mutants compared to control worms after infection of *D. coniospora* and treatment with spiny balls. Similarly, mutations in *egl-30* or *egl-8* significantly suppressed the expression of *Psod-3::GFP* induced by *Drechmeria coniospora* and spiny balls (Figure 5C).

Both *egl-30(n686)* and *egl-8(n488)* mutants were more sensitive than wild-type worms to killing by *D. coniospora* (Figure 5D) or spiny balls (Figure 5E), respectively. However, mutations in *egl-30* and *egl-8* did not alter the *daf-16(mu86)* phenotype. *daf-16(mu86); egl-30(n686)* or *daf-16(mu86); egl-8(n488)* double mutants were indistinguishable from *daf-16(mu86)* for sensitivity to *D. coniospora* (Figure 5D) or spiny balls (Figure 5E), suggesting that these genes function in a common pathway. In *C. elegans*, *egl-30* is expressed in many tissues, including the epidermis [42]. In contrast, *egl-8*, which is predominantly expressed in neurons, has been shown to act genetically downstream of *egl-30* [43]. We found that epidermal-specific, rather than intestinal-specific, knock-down of *egl-30* or *egl-8* significantly reduced the survival rate of nematodes after *D. coniospora* infection and treatment with spiny balls (Figure S13A–D in Text S1). In addition, epidermal-specific expression of *egl-30* or *egl-8* was sufficient to rescue immune-deficient phenotypes in *egl-30(n686)* and *egl-8(n488)* mutants to *D. coniospora* infection and physical injury, respectively (Figure S14A and S14B in Text S1). These results suggest that the EGL-30-EGL-8 pathway functions within the epidermis to promote innate immunity against fungal challenge and wound response.

### CST acts downstream of BLI-3 to regulate the nuclear accumulation of DAF-16

**Figure 6:**
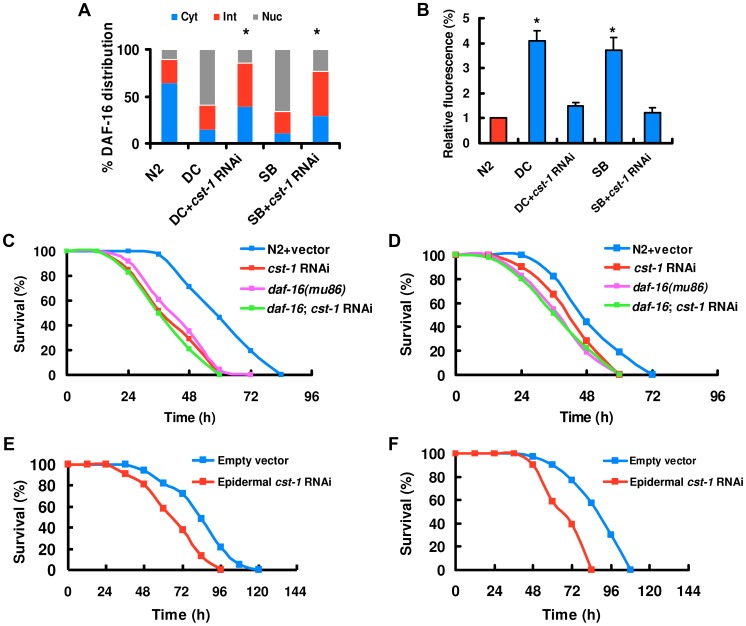
CST regulates the nuclear accumulation of DAF-16. (A) *cst-1* RNAi diminished DAF-16 nuclear translocation by *D. coniospora* infection (DC) and treatment with spiny balls (SB). n = 100–110 nematodes per condition. These results are means±SD of four experiments. *P<0.05 *versus* control (DC and SB). (B) Knock-down of *itr-1* inhibited the expression of *Psod-3::GFP* induced by *D. coniospora* infection and spiny balls for 12 h. *P<0.05 *versus* control (N2). (C and D) *cst-1* RNAi reduced the survival rate of nematodes exposed to *D. coniospora* (C) and spiny balls (D). *P*<0.001 relative to control with empty vector. However, *cst-1* RNAi did not enhanced susceptibility of *daf-16(mu86)* mutants to killing by *D. coniospora* (C) and spiny balls (D). (E and F) Epidermal-specific RNAi of *cst-1* significantly reduced the survival rate of worms exposed to *D. coniospora* (E) and spiny balls (F). *P*<0.001 relative to control with empty vector.

It has been reported that the mammalian Ste20-like kinase-1 (MST1) mediates oxidative stress-induced activation of FOXO transcription factors [44]. In *C. elegans*, CST-1, the ortholog of mammalian MST1, promotes life-span extension in a DAF-16-dependent manner [44]. Thus, we hypothesized that CST-1 might function analogously to MST1 as an activator of DAF-16. To test this idea, we assayed the effect of *cst-1* knock-down on DAF-16 activation by induced by *D. coniospora* and spiny balls. *cst-1* knock-down by RNAi led to a significant reduction in *cst-1* expression (Figure S15 in Text S1). *cst-1* RNAi significantly suppressed the nuclear accumulation of DAF-16 (Figure 6A), but did not influence the production of ROS induced by *D. coniospora* and spiny balls (Figure S16 in Text S1). Similarly, knock-down of *cst-1* by RNAi significantly inhibited the expression of *Psod-3::GFP* induced by *D. coniospora* and spiny balls (Figure 6B). These results suggest that CST-1 acts upstream of DAF-16, but downstream of BLI-3 in response to fungal infection and wounding.

Knock-down of *cst-1* by RNAi reduced the survival of nematodes after *D. coniospora* infection (Figure 6C) and treatment with spiny balls (Figure 6D). However, the survival of *daf-16(mu86);cst-1* RNAi was comparable to that of *daf-16(mu86)* mutants (Figure 6C and 6D). These data suggest that *daf-16* is epistatic to *cst-1*. *cst-1* is mainly expressed in the epidermis, tail, vulva, and sensory neurons in the head [44]. We found that epidermal-specific *cst-1* RNAi resulted in enhanced sensitivity after *D. coniospora* infection (Figure 6E) and treatment with spiny balls (Figure 6F), whereas intestinal-specific *cst-1* RNAi did not affect the survival of worms (Figure S17A and S17B in Text S1). These results indicate that *cst-1* is required for innate immunity in the epidermis.

A previous study demonstrated that BAR-1, the ortholog to mammalian β-catenin, is required for oxidative stress-induced DAF-16 activity in *C. elegans* [33]. BAR-1 also plays a positive role in *C. elegans* intestinal immunity to *S. aureus* [45]. Thus, *bar-1* might be expected to act genetically downstream of *bli-3* to activate DAF-16. However, the nuclear accumulation of DAF-16 was not altered in the *bar-1(ga80)* mutants after *D. coniospora* infection and treatment with spiny balls (Figure S18 in Text S1). These results suggest that BAR-1 is not involved in the activation of DAF-16 upon fungal infection and wound response.

## Discussion

In a variety of animals, the epidermis may represent a first line of defense against pathogenic infection and physical injury. The key finding in this study is that the DAF-16/FOXO transcription factor is a direct regulator of immune responses associated with epidermal damage. Our data also provides a novel molecular mechanism by which DAF-16 is activated by pathogenic fungi and wounding, and that the pathway is independent of the DAF-2 insulin-like signaling pathway.

A sustained production of ROS following injury has been observed in human cells, the zebrafish and *Drosophila* tissues [30], [31], [32]. Recent studies demonstrate that these processes are mediated by DUXOs [31], [32]. In the current study, we observe that ROS production is mediated by BLI-3 in the epidermis after fungal infection and physical injury. Our study indicates that ROS production is crucial for resistance to fungal infection and physical injury in *C. elegans*, supporting the idea that injury-induced ROS production is an important regulator of tissue regeneration [46]. Furthermore, our results demonstrate that ROS production is required for activation of DAF-16, which, in turn is essential for resistance to fungal infection and physical injury in *C. elegans*. Two recent studies indicate that knock-down of *bli-3* by RNAi leads to enhanced susceptibility to *E. faecalis* [39], [47]. However, the *daf-16* mutants exhibit a comparable degree of susceptibility to *E. faecalis*-mediated killing as wild-type worms [4], [48]. These results indicate that the protective effect of BLI-3 on *E. faecalis* infection is not mediated through DAF-16.

Impairment in release of Ca^2+^ abolished ROS production upon fungal challenge and wounding, suggesting that BLI-3 enzymatic activity is dependent on Ca^2+^. The EF-hand calcium-binding motif in *C. elegans* BLI-3 has a relatively low amino acid identity (41%) and similarity (61%) to the human DUOX1, casting doubt as to whether calcium binding is required for *C. elegans* BLI-3 function [38]. However, the EF-hand calcium-binding motif in *Drosophila* DUOX1 also shares a relatively low identity (42%) and similarity (66%) to the human DUOX1. Because ROS-producing *Drosophila* DUOX1 enzymatic activity depends on intracellular Ca^2+^ through binding to the EF-hand domains [49], [50], it is plausible that Ca^2+^ modulates the enzymatic activity of *C. elegans* BLI-3.

The PI3K-Akt-FOXO signaling pathway is evolutionarily conserved from nematodes to mammals [10], [51]. In mammalian cells, protein kinase Akt, a downstream effector of the insulin-signaling pathway, phosphorylates two sites (Thr32 and Ser252) on the FOXO3 protein leading to its nuclear exclusion and inactivation [52]. Likewise, *P. aeruginosa* suppresses the activity of DAF-16 by activating DAF-2 insulin-like signaling [7], [26]. However, our data demonstrate that causal involvement of diminished DAF-2 insulin-like signaling in the activation of DAF-16 by fungal infection is unlikely, suggesting that alternative mechanisms are involved. A previous study has demonstrated that oxidative stress activates FOXO3 through an MST1-mediated mechanism [44]. Under oxidative stress, MST1 phosphorylates FOXO3 at Ser207 and the phosphorylation of FOXO3 in turn induces its dissociation from 14-3-3 proteins and translocation to the nucleus [44]. Although knock-down of *daf-16* by RNAi completely inhibits the ability of CST-1 to extend life span in *C. elegans*, whether CST-1 activates DAF-16 under oxidative stress remains unclear. Our data indicate that ROS mediates activation of DAF-16 in response to epidermal damage in a CST-dependent manner. These results support a model in which the evolutionarily conserved MST/CST pathway functions in parallel with the insulin signaling pathway to regulate FOXO/DAF-16 by oxidative stress [44].

The epidermis forms a protective barrier against physical damage and pathogen entry [53], [54]–[55]. An intimate relationship between wound repair and innate immunity is widely accepted [56]. Previous studies have shown that epidermal immune responses to fungal infection and physical wounding share some of the same signals and mediators in *C. elegans* 19,24. For instance, Gα12/GPA-12 acts, together with the two phospholipases EGL-8 and PLC-3, upstream of the PKC-TIR-1-p38 MAPK pathway, to induce a set of the *nlp* genes encoding antimicrobial peptides (AMPs) in response to fungal challenge and needle wounding [19], [24]. A recent study has shown that the EGL-30-EGL-8 signaling pathway triggers epidermal Ca^2+^ release through IP3 and its receptor ITR-1 after wounding [29]. In this study, our results indicate that DAF-16 is activated by EGL-30-Ca^2+^ upon fungal infection and physical injury. Since epidermal DAF-16 is required for innate immune response to fungal infection and physical injury, it is an important immune effector of EGL-30-Ca^2+^ in the epidermis. However, mutations in *daf-16* do not alter the expression of AMPs induced by fungal infection, which is consistent with the observation that the EGL-30-Ca^2+^ pathway appears not to be involved in the up-regulation of AMPs after wounding [29].

Because FOXOs are conserved from worms to humans, it is of great interest to investigate whether FOXOs are involved in epidermal innate immunity in other species (e.g., humans). FOXOs have been shown to mediate the induction of antimicrobial peptides, such as defensins, both in *Drosophila* and human tissues [10]. Accumulating evidence indicates that defensins, the major skin-derived antimicrobial peptides, not only act as endogenous antibiotics, but also participate in additional roles such as promoting wound repair [57], [58]. Meanwhile, inhibition of PI3K, a component of insulin/insulin-like growth factor signaling, by LY294002 (a specific inhibitor of PI3K), strongly accelerates scratch closure in human keratinocytes [59]. Because reduced signaling of the insulin/insulin-like growth factor pathway leads to the activation of FOXO transcription factors, these results imply that FOXOs are probably involved in keratinocyte wound healing. A recent study has investigated epidermal gene expression in wounded skin from three donors and examined transcription factor binding sites (TFBS) in the promoters of the 100 most differentially expressed genes [60]. Highly significant overrepresentations of TFBS for FOXO transcription factors are identified. These data suggest that FOXOs are possibly involved in controlling the epidermal gene expression during the proliferative phase of wound healing. Thus, the DAF-16/FOXO transcription factor that functions as an effector of innate immunity in epidermal tissues seems to be evolutionarily conserved in various animal species including worms, insects and mammals.

**Figure 7:**
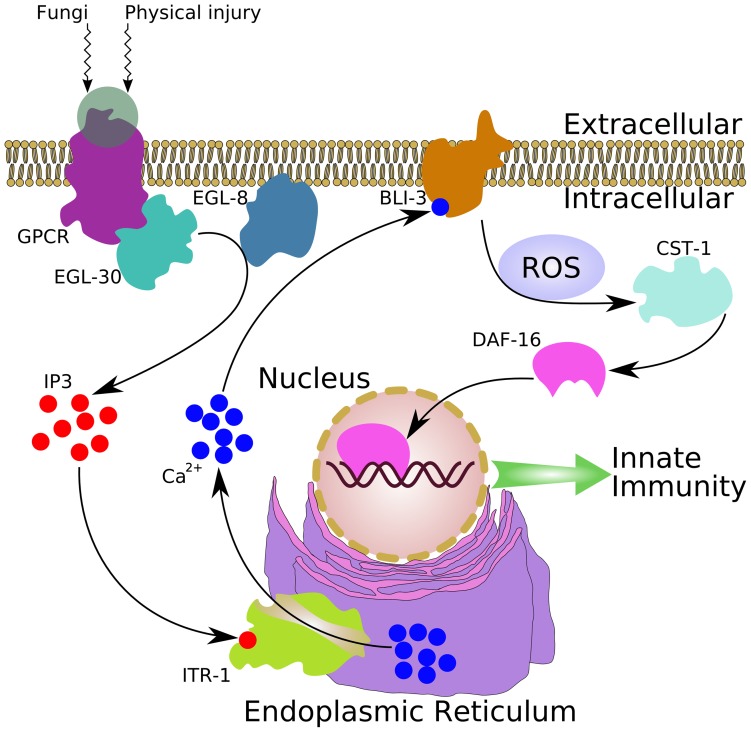
A proposed mechanism of *C. elegans* DAF-16 activation by fungal infection and physical injury. Fungal infection or physical injury in the epidermis activates an unknown G protein-coupled receptor (GPCR), which in turn activates EGL-30 (Gαq). EGL-30 may positively modulate EGL-8 (PLCβ) activity, resulting in the production of inositol 1,4,5-trisphosphate (IP3). IP3 then binds to the IP3 receptor ITR-1 located in the endoplasmic reticulum membrane to initiate the release of Ca^2+^ stored in this organelle. The released Ca^2+^ may activate BLI-3 activity through its Ca^2+^-sensitive EF hand domain to produce ROS. Subsequently, ROS induces the CST-mediated accumulation of DAF-16 in the nucleus to promote innate immunity against fungal infection and physical injury.

In summary, our findings suggest that DAF-16 is directly involved in innate immunity in the epidermis. EGL-30/Ca^2+^/BLI-3/ROS/CST-1 signaling represents a novel pathway to regulate DAF-16 activity (see model in Figure 7), which is functionally independent of the DAF-2 insulin-like signaling pathway. Based on these findings, we propose that FOXO/DAF-16 could be a novel target for the treatment of epidermal damage.

## Materials and Methods

### Nematode strains

The following *C. elegans* strains were used in this study: N2 (wild-type), *daf-16(mu86)*, *bli-3(e767)*, *itr-1(sa73)*, *bar-1(ga80)*, *egl-30(n686)*, *egl-8(n488)*, *ins-7(ok1573)*, TJ356*-daf-16::gfp(zIs356 (pDAF-16::DAF-16-GFP;rol-6))*, *muIs84 (Psod-3::gfp)*, NR222 *(rde-1(ne219); kzIs9[pKK1260(plin-12::nls::gfp)*, *pKK1253(plin-26::rde-1)*, *rol-6])*; and NR350 *(rde-1(ne219); kzIs20[pDM#715(phlh-1::rde-1), pTG95(psur-5::nls::GFP), rol-6])* were kindly provided by the Caenorhabditis Genetics Center (CGC). The CZ13896 (*Pcol-19-GCaMP (juIs319)*), CZ12690 (*Pcol-19-Superspronge (juEx3052)*), and CZ15386 (*egl-8(sa47V;egl-8(juEX4257)*) strains were kindly provided by Dr. Andrew D. Chisholm (University of California San Diego). The strain GR1353 (*daf-2(e1370) III; mgIs41[daf-16::gfp]*) and the strain for intestinal-specific RNAi *(sid-1(qt9); Is[vha-6pr::sid-1]; Is[sur-5pr::GFPNLS])* were kindly provided by Dr. Gary Ruvkun (Massachusetts General Hospital, Harvard Medical School).

Mutants and transgenic strains were backcrossed three times into the N2 strain used in the laboratory. All strains were maintained on nematode growth media (NGM) and fed with E. coli strain OP50.

### Infection with fungi and bacteria

Standard conditions were used for *C. elegans* growth at 20°C [61]. Synchronized populations of worms were cultivated at 20°C until the mid-L4 stage. For all pathogen assays, 75 µg/ml of fivefluoro-2′-deoxyuridine (FUdR) was added to the assay plates to abolish the growth of progeny.

Killing assays with *D. coniospor*a: 50–60 L4 nematodes were transferred to fresh plates seeded with heat-killed *E. coli* OP50, with ∼1.0×10^8^ *D. coniospora* spores at 25°C. Killing assays with *C. rosea*: ∼1.0×10^8^ spores of *C. rosea* were inoculated onto plates containing heat-killed *E. coli* OP50 for 1–2 days at 28°C, and the infection experiments were started by adding 50–60 nematodes to each plate at 25°C. The number of living worms was counted by using a light microscope at time intervals. Immobile nematodes unresponsive to touch were scored as dead. One-sided rank log tests were used to the statistical significance of the differences between treatments.

Killing assays with *P. aeruginosa*: *P. aeruginosa* PA14 (a gift from Dr. Kun Zhu, Institute of Microbiology, CAS) was cultured in Luria broth (LB), then seeded on slow-killing plates, which contain modified NGM (0.35% instead of 0.25% peptone). PA14 was incubated first for 24 h at 37°C and then for 24 h at 25°C. The infection experiments were started by adding 50–60 nematodes to each plate at 25°C. Killing assays with *S. aureus*: *S. aureus* ATCC 25923 (a gift from Dr. Wen-Hui Lee, Kunming Institute of Zoology, CAS) was cultured in tryptic soy broth (TSB, BD, Sparks, MD), then seeded on plates containing modified NGM (0.35% instead of 0.25% peptone). The infection experiments were started by adding 50–60 nematodes to each plate at 25°C.

### Physical injury by spiny balls

Spiny balls were purified by a previously described method [25]. The spiny ball suspension was adjusted to ∼1.0×10^5^ per ml. 100 µl of the spiny ball suspension was thoroughly added to on plates containing modified NGM with heat-killed *E. coli* OP50. The infection experiments were started by adding 50–60 nematodes to each plate at 25°C. Mobile and immobile nematodes were counted every 12 h after their addition.

### RNA interference

RNAi bacterial strains containing targeting genes were obtained from the Ahringer RNAi library [62]. RNAi feeding experiments were performed on synchronized L1 larvae at 20°C for 40 h. L4 larvae or young adult worms were used in immunity assays. The strain NR222 was used in epidermis-specific RNAi, the strain *(sid-1(qt9); Is[vha-6pr::sid-1]; Is[sur-5pr::GFPNLS])* was used in intestine-specific RNAi , and the strain NR350 was used in muscular-specific RNAi.

### DAF-16 nuclear localization assay

After 12 h of fungal infection or treatment with spiny balls, the worms were immediately mounted in M9 onto microscope slides. The slides were viewed using a Zeiss Axioskop 2 plus fluorescence microscope (Carl Zeiss, Jena, Germany) with a digit camera. The status of DAF-16 localization was categorized as cytosolic localization, nuclear localization when localization is observed throughout the entire body from head to tail, or intermediate localization when there is a visible nuclear localization but one not as complete as nuclear [63]. The number of worms with each level of nuclear translocation was counted.

### Quantitative real-time RT-PCR analysis

Total RNA from worms was isolated using Trizol reagent (Invitrogen, Carlsbad, CA). Random-primed cDNAs were generated by RT of the total RNA samples using a standard protocol. A real-time-PCR analysis was performed with the ABI Prism 7000 Sequence Detection system (Applied Biosystems, Foster City, CA) using SYBR Premix-ExTag (Takara, Dalian, China). β-Tubulin was used for internal control. The primers used for PCR are listed in Table S2.

### Construction of transgenes

The *dpy-7* and *clo-19* genes have shown to be expressed in the epidermis [29]. The *Pdpy-7:daf-16* fusion gene was chemically synthesized, and obtained from Generay Biotech Co. (Shanghai, China). The DNA fragment contains a 436 bp of *dpy-7* promoter fragment (corresponding to nucleotide −436 to −1 relative to the translational start site), a 1530 bp of the *daf-16* cDNA, a 729 bp of the GFP cDNA and a 234 bp of the 3′-UTR of *unc-5*4. The *Pcol-19:egl-30* fusion gene was constructed as follows. A 2838 bp of *col-19* promoter fragment was obtained by PCR on *C. elegans* genomic DNA using primers 5′-GCT CTA GAG CAT CGT CAC ATT CTG TCT-3′ and 5′-TCC CCC GGG GGC TTT CCA TCG TCT CC-3′ followed by XbaI and SmaI digestion. The fragment was inserted into XbaI and SmaI digested pPD95.79, resulting in plasmid pPDegl. A 1116 bp fragment of *egl-30* cDNA was amplified by PCR on *C. elegans* genomic DNA using primers 5′- TCC CCC GGG TTG TTC TAT TCG CTG GCT T-3′ and 5′-GGG GTA CCC CAA GTT GTA CTC CTT CAG ATT AT-3′ followed by SmaI and KpnI digestion. The fragment was inserted into SmaI and KpnI digested pPDegl vector.

The *Pdpy-7:daf-16* fusion gene fragment or the vector containing *Pcol-19::egl-30* fusion gene was co-injected with *rol-6* plasmid (pRF4) into gonads of wild-type and *egl-30(n686)* animals by standard techniques [64]. The transgenic worms carrying *Pdyp-7::daf-16* or *Pcol-19::egl-30* were confirmed in prior to each pathogenesis assay.

### Measurement of ROS

The ROS levels were detected by 2′,7′-dichlorodihydrofluorescein diacetate (DCF-DA) as a probe as described previously with modifications [34], [35], [36]. Briefly, after infected with pathogens or treated with spiny balls for 8 h, about 1000 worms from each group were collected in M9 buffer and washed three times to eliminate conidia. Then, the worms were transferred to a 1.5-mL tube containing 150 µl PBS with 1% Tween 20, and immediately frozen in liquid nitrogen. After thawing at room temperature, the worms were subjected to sonication (Branson Sonifier 250; VWR Scientific, Suwanee, GA). Samples were vortexed, and supernatants were collected after centrifugation. The supernatant containing 10 µg protein was transferred into 96-well plates, and incubated with 15 µL of 100 µM DCF-DA in PBS at 37°C in a Spectra Max M5 fluorescent microplate reader (Molecular Devices, Sunnyvale, CA) for quantification of fluorescence at excitation 485 nm and emission 530 nm. Samples were read kinetically every 20 min for 2.5 h.

### Analysis of Ca^2+^ in the epidermis using GCaMP fluorescence

To analyze Ca^2+^ in the epidermis, GCaMP fluorescence imaged was obtained using confocal microscopy (Zeiss LSM-510) with a 40×objective, as described previously [29]. Briefly, average fluorescence was determined in ten equivalent regions of interest (ROI), five centered on the epidermal cell and five in the background. Baseline fluorescence (F_0_) and induction fluorescence (Ft) were obtained by averaging fluorescence in five ROIs in the epidermis then subtracting the average of five ROIs in the background before and after fungal infection or injury. GCaMP fluorescence was normalized to an internal control, *Pcol-19*-tdTomato. The change in fluorescence ΔF was expressed as the ratio of change with respect to the baseline [(Ft–F_0_)/F_0_]. Raw data from fluorescent microscopy were then analyzed using ImageJ.

### Statistical analysis

Differences in survival rates were analyzed using the log-rank test. Differences in gene expression, distribution of DAF-16, and fluorescence intensity were assessed by performing a one-way ANOVA followed by a Student-Newman-Keuls test. Data were analyzed using SPSS11.0 software (SPSS Inc.). To test for significant overlap between different gene lists, a Fisher's exact test was used.

## Supporting Information

Table S1 **The target genes of DAF-16 up-regulated by** ***D. coniospora*** **infection.** When compared DAF-16 target genes to published microarray analysis of gene expression induced by *D. coniospora* infection, 48 of the genes up-regulated by *D. coniospora* were found to be targets of DAF-16.
(DOC)

Table S2 **The primers are used for real-time PCR.** This table lists all primers for real-time PCR analysis.
(DOC)

Text S1 **Supporting Figures. Figure S1. Fungal infection and mutation in daf-12 induce DAF-16 nuclear translocation.** (A) Wild-type worms in the absence of *D. coniospora*. (B) Wild-type worms were exposed to *D. coniospora* for 12 h. (C) *daf-2* mutants were growth under normal conditions without *D. coniospora*. **Figure S2. Both epidermal- and intestinal-specific knock-down of** ***daf-16*** **by RNAi suppress the expression of DAF-16 target genes.** (A) qPCR analysis of expression of DAF-16 target genes in NR222 strains (CTR), CTR 24 h after *D. coniospora* infection (CTR+DC), and CTR subjected to *daf-16* RNAi after *D. coniospora* infection (CTR+DC+epidermal *daf-16* RNAi). (B) qPCR analysis of expression of the intestinal-specific RNAi strain *sid-1(qt9);Is[sur-5::GFP]; alxIs7[VHA-6p::SID-1::SL2::GFP]* (CTR), CTR 24 h after *D. coniospora* infection (CTR+DC), and CTR subjected to *daf-16* RNAi after *D. coniospora* infection (CTR+DC+epidermal *daf-16* RNAi). *P<0.05, CTR+DC relative to CTR+DC+ *daf-16* RNAi. **Figure S3. Genetic loss of** ***ins-7*** **has no effect on DAF-16 translocation and the survival of worms after** ***D. coniospora*** **infection and physical injury.** (A) Mutation in *ins-7(ok1573)* did not influence the nuclear accumulation of DAF-16 after *D. coniospora* infection (DC) and treatment with spiny balls (SB). (B and C) Mutation in *ins-7(ok1573)* did not affect the survival of nematodes after *D. coniospora* infection (B) and treatment with spiny balls (C). **Figure S4. DAF-16 is required for resistance to fungal infection.** (A) *daf-16(mu86)* mutants were sensitive to *C. rosea* infection. (B–C) *daf-16* RNAi reduced the survival rate of nematodes exposed to *D. coniospora* (B) and *C. rosea* (C). *P*<0.001 relative to wild-type animals. **Figure S5. Epidermal-specific knock-down of** ***daf-16*** **reduces the expression of** ***daf-16*** **in the hypodermis.** (A) NR222 strains were exposed to *D. coniospora* for 12 h. (B) NR222 strains subjected to *daf-16* RNAi were exposed to *D. coniospora* for 12 h. **Figure S6. Muscular-specific** ***daf-16*** **RNAi has no effect on sensitivity to** ***D. coniospora*** **infection and spiny balls.** (A) NR350 strains were exposed to *D. coniospora*. (B) NR350 strains were exposed to spiny balls. After 12 h of treatment, the DAF-16::GFP expression pattern was observed. **Figure S7. Elevated** ***daf-16*** **expression in the epidermis confers resistance to fungal infection and physical injury.** Overexpression of *daf-16* under epidermal (*dpy-7*) promoter increased survival of nematodes after *D. coniospora* infection (A) and treatment of spiny balls (B). **Figure S8. Knock-down of** ***bli-3*** **in a 1/10 dilution results in a decrease in the expression of** ***bli-3*** **.** qPCR analysis of *bli-3* expression in WT worms subjected to *bli-3* RNAi in a 1/10 dilution. **Figure S9. BLI-3 in the epidermis is required for ROS production, DAF-16 nuclear accumulation, and resistance to fungal infection and physical injury.** (A) Epidermal-specific RNAi of *bli-3* in a 1/10 dilution significantly reduced the production of ROS induced by *D. coniospora* (DC) or spiny balls (SB). (B) Epidermal-specific RNAi of *bli-3* in a 1/10 dilution significantly reduced DAF-16 nuclear accumulation induced by *D. coniospora* or spiny balls. (C–D) Epidermal-specific RNAi of *bli-3* in a 1/10 dilution significantly reduced the survival rate of nematodes exposed to *D. coniospora* (C) or spiny balls (D). (E–F) Intestinal-specific RNAi of *bli-3* in a 1/10 dilution did not influence the survival of nematodes after *D. coniospora* infection (E) and treatment with spiny balls (F). **Figure S10. The peroxidase activity of BLI-3 is not crucial for resistance to fungal infection and physical injury**. (A–B) The mutant *bli-3(e767)* encoding a protein that lacks the peroxidase domain exhibited similar sensitivity to killing by *D. coniospora* infection (A) and spiny balls (B) as wild-type animals. **Figure S11. A disturbance of IP3 signaling reduces the survival of worms after fungal infection and physical injury.** (A–B) Worms overexpressing IP3 sponges (cz12690) in the epidermis exhibited enhanced susceptibility to killing by *D. coniospora* (A) and spiny balls (B). **Figure S12. ITR-1 in the epidermis is required for resistance to fungal infection and physical injury.** (A–B) Epidermal-specific RNAi of *itr-1* significantly reduced the survival rate of worms after *D. coniospora* infection (A) or treatment with spiny balls (B). (C–D) Intestinal-specific knock-down of *itr-1* did not influence the survival of nematodes after *D. coniospora* infection (C) and treatment with spiny balls (D). **Figure S13. Epidermal-specific, rather than intestinal-specific, knockdown of** ***egl-30*** **or** ***egl-8*** **suppresses innate immunity.** (A–B) Epidermal-specific knock-down of *egl-30* or *egl-8* by RNAi reduced the survival of nematodes exposure to *D. coniospora* infection (A) and spiny balls (B). (C–D) Intestinal-specific knock-down of *egl-30* or *egl-8* did not influence the survival of nematodes after *D. coniospora* infection (C) and treatment with spiny balls (D). **Figure S14. EGL-30 and EGL-8 in the epidermis are required for resistance to fungal infection and physical injury.** (A–B) Epidermal-specific expression of *egl-30* rescued immune-deficient phenotypes in *egl-30(n686)* mutants to *D. coniospora* infection (A) and treatment with spiny balls (B). (C–D) Epidermal-specific expression of *egl-8* (cz15896) rescued immune-deficient phenotypes in *egl-8(n488)* mutants to *D. coniospora* infection (C) and treatment with spiny balls (D). **Figure S15.** ***cst-1*** **RNAi significantly reduces the expression of** ***cst-1*** **.** After worms were subjected to *cst-1* RNAi,the expression of *cst-1* was determined by qPCR. **Figure S16. CST is not required for ROS production induced by fungal infection and physical injury.** *cst-1* RNAi did not influence the production of ROS induced by *D. coniospora* (DC) and spiny balls (SB). **Figure S17. CST-1 in the intestine is not required for resistance to fungal infection and physical injury.** (A–B) Intestinal-specific knock-down of *cst-1* by RNAi did not affect the survival rate of nematodes after *D. coniospora* infection (A) and treatment with spiny balls (B). **Figure S18. BAR-1 is not involved in DAF-16 activation upon fungal infection and physical injury.** Mutations in *bar-1(ga80)* did not influence the nuclear accumulation of DAF-16 after *D. coniospora* infection (DC) and treatment with spiny balls (SB).
(PDF)
