# Supplementary material for: The DAF-16/FOXO Transcription Factor Functions as a Regulator of Epidermal Innate Immunity
Source: PLoS Pathog. 2013 Oct 17;9(10):e1003660. doi: 10.1371/journal.ppat.1003660 (PMC3798571; doi:10.1371/journal.ppat.1003660)
Supplement: Table S1 — The target genes of DAF-16 up-regulated by D. coniospora infection. When compared DAF-16 target genes to published microarray analysis of gene expression induced by D. coniospora infection, 48 of the genes up-regulated by D. coniospora were found to be targets of DAF-16. (DOC) [file ppat.1003660.s001.doc]

**Table S1. The target genes of DAF-16 up-regulated by *D. coniospora* infection**

| **Gene ID** | **Gene Name** | **Brief description** |
| --- | --- | --- |
| B0213.15 | cyp-34A9 | Member of the cytochrome P450 family |
| C08E3.6 | C08E3.6 | Protein containing an F-box domain, which serve as a link between a target protein and a ubiquitin-conjugating enzyme |
| C15H9.1 | nnt-1 | encodes a proton-pumping nicotinamide nucleotide transhydrogenase predicted to be mitochondrial. |
| C18A11.1 | C18A11.1 | Protein of unknown function, has weak similarity to C. elegans T24C4.3 |
| C24B9.9 | dod-3 | Downstream Of DAF-16 (regulated by DAF-16) |
| C25E10.9 | swm-1 | encodes a putative secreted TIL-domain protease inhibitor |
| C34C6.7 | C34C6.7 | unknown |
| C45G7.3 | ilys-3 | Invertebrate LYSozyme |
| C50F7.5 | C50F7.5 | unknown |
| C52E4.1 | cpr-1 | encodes a cysteine protease of the cathepsin B-like cysteine protease family |
| C54C8.9 | nlp-39 | Neuropeptide-Like Protein |
| CC8.2 | CC8.2 | unknown |
| C02A12.4 | lys-7 | encodes an enzyme homologous to an antimicrobial lysozyme |
| F08B12.4 | F08B12.4 | unknown |
| F09F7.6 | F09F7.6 | unknown |
| F11A5.12 | stdh-2 | encodes a putative steroid dehydrogenase required for normally short lifespan; |
| F12A10.7 | F12A10.7 | unknown |
| F15A2.2 | tre-4 | TREhalase |
| F15A4.8 | F15A4.8 | unknown |
| F15B9.1 | far-3 | Fatty Acid/Retinol binding protein |
| F15E6.4 | F15E6.4 | unknown |
| F15E6.8 | dct-7 | DAF-16/FOXO Controlled, germline Tumor affecting |
| F28D1.3 | thn-1 | THaumatiN family |
| F28D1.5 | thn-2 | THaumatiN family |
| F36F2.2 | F36F2.2 | unknown |
| F38E11.2 | hsp-12.6 | Member of the Hsp20 or alpha crystallin family |
| F40D4.3 | srh-159 | Serpentine Receptor, class H |
| F45D3.4 | F45D3.4 | unknown |
| F46C5.1 | F46C5.1 | Protein of unknown function |
| F47B8.4 | F47B8.4 | Protein with strong similarity to C. elegans F47B8.3 |
| F53A9.1 | F53A9.1 | Protein of unknown function |
| K01A2.2 | far-7 | Fatty Acid/Retinol binding protein |
| K07E3.3 | dao-3 | encodes a protein containing tetrahydrofolate dehydrogenase/cyclohydrolase catalytic and NAD(P)-binding domains |
| K11G9.6 | mtl-1 | encodes one of two C. elegans metallothioneins, small, cysteine-rich, metal-binding proteins; |
| K12G11.3 | sodh-1 | Sorbitol DeHydrogenase family |
| R05F9.13 | msp-31 | encodes a member of the major sperm protein family |
| T07H3.3 | math-38 | MATH (meprin-associated Traf homology) domain containing |
| T19B10.2 | T19B10.2 | unknown |
| T21C9.8 | ttr-3 | TransThyretin-Related family domain |
| T22F3.11 | T22F3.11 | Protein of unknown function |
| T28F4.5 | T28F4.5 | gene encodes a homolog of Death Associated Protein 1 (DAP-1) protein that may be involved in apoptosis. |
| W10G6.3 | mua-6 | encodes an essential intermediate filament protein (MUA-6/IFA-2) |
| Y40B10A.6 | comt-4 | Catechol-O-MethylTransferase family |
| Y43C5A.3 | Y43C5A.3 | unknown |
| Y46C8AR.1 | clec-76 | C-type Lectin |
| Y51A2D.11 | ttr-26 | TransThyretin-Related family domain |
| ZC395.5 | ZC395.5 | unknown |
| Y40B10A.6 | Y40B10A.6 | Member of the type 3 O-methyltransferase family, has high similarity to uncharacterized C. elegans Y32B12A.3 |
