# Supplementary material for: The DAF-16/FOXO Transcription Factor Functions as a Regulator of Epidermal Innate Immunity
Source: PLoS Pathog. 2013 Oct 17;9(10):e1003660. doi: 10.1371/journal.ppat.1003660 (PMC3798571; doi:10.1371/journal.ppat.1003660)
Supplement: Table S2 — The primers are used for real-time PCR. This table lists all primers for real-time PCR analysis. (DOC) [file ppat.1003660.s002.doc]

**Table S2. The primers are used for real-time PCR.**

| **Genes** | **Sequences (5’→3’)** | **Product length (bp)** |
| --- | --- | --- |
| *thn-1* forward:  reverse: | CAAGTGCCCATTCACCATC  CGTTACATTGCTCCGAGTTTCT | 200 |
| *tre-4* forward:  reverse: | GGACCTCATTTGGACACTTAGA  GGCTTCTTCATCATCCCGTT | 190 |
| *lys-7* forward:  reverse: | ATGACTCCACAGCCCGTTT  AGTTGGCGAAGTGACCTGA | 120 |
| *thn-2* forward:  reverse: | AGGAGGCTTCCAACTTACGG  GTTGCATTGCTCCGAGTTTC | 145 |
| *B0213.15* forward:  reverse: | CACAGAGTTCCGACCTGAAA  AGCCAGTGATTCGCCAAGA | 103 |
| *F15A4.8* forward:  reverse: | TGGAGTTTGGATTTGGACAG  TAGATTCAACAGGAGGCGTAGT | 137 |
| *C25E10.9* forward:  reverse: | GTGAAGCAACTTGTGAGAAACC  CGAAACCTTTGGAGCATTG | 84 |
| *F53A9.1* forward:  reverse: | CGGAAACGGAGGATACAA  GATGATGATGGTGCCCA | 119 |
| *ins-7* forward:  reverse: | CCCTGCTTCTCAACAATA  CTTCTTCCACAACGATAAAT | 153 |
| *ins-1* forward:  reverse: | CGTCTCACAACAACCCTT  CGCTTCTCACAACATTCTG | 175 |
| *bli-3* forward:  reverse: | TCTTTCAAACAAGGGCGG CCTGGATTCTCATTCACACG | 161 |
| *cst-1* forward:  reverse: | GATTCCAACTAAACCACCACCT TCATTTCCGCTGCTTGCT | 239 |
| *β-actin* forward:  reverse: | CCATCATGAAGTGCGACATTG  CATGGTTGATGGGGCAAGAG | 134 |
