# Supplementary material for: The DAF-16/FOXO Transcription Factor Functions as a Regulator of Epidermal Innate Immunity
Source: PLoS Pathog. 2013 Oct 17;9(10):e1003660. doi: 10.1371/journal.ppat.1003660 (PMC3798571; doi:10.1371/journal.ppat.1003660)

Supplemental Information for

The DAF-16/FOXO transcription factor functions as a regulator of epidermal innate immunity

Cheng-Gang Zou, Qiu Tu, Jie Niu, Xing-Lai Ji, and Ke-Qin Zhang

Figure S1

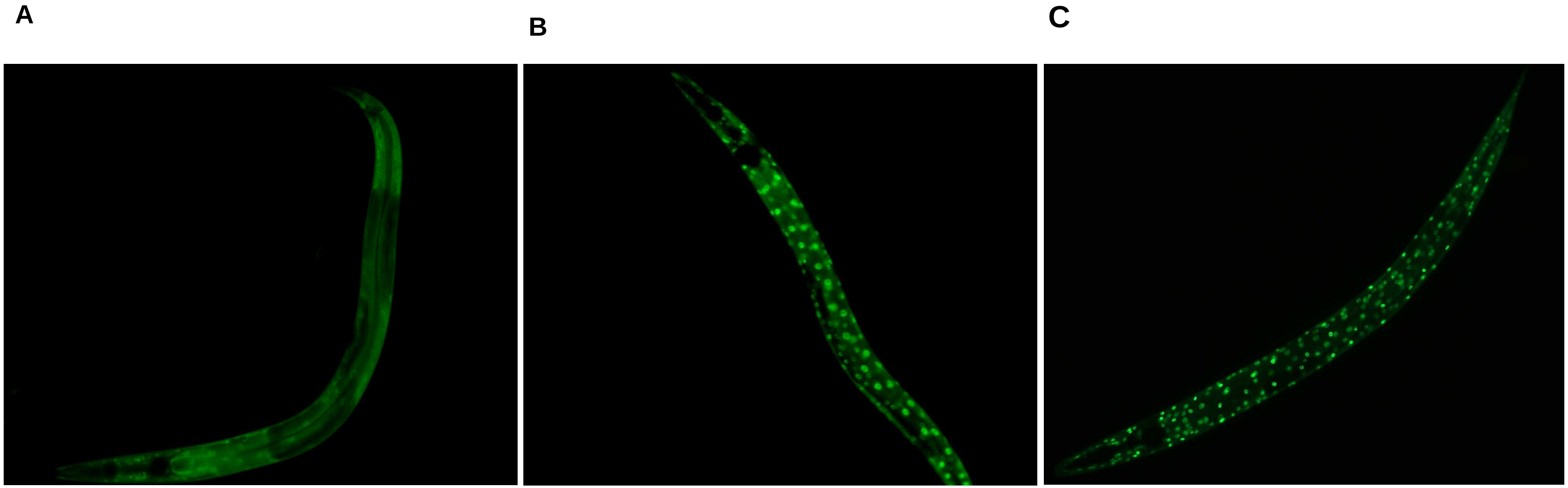

Figure S2

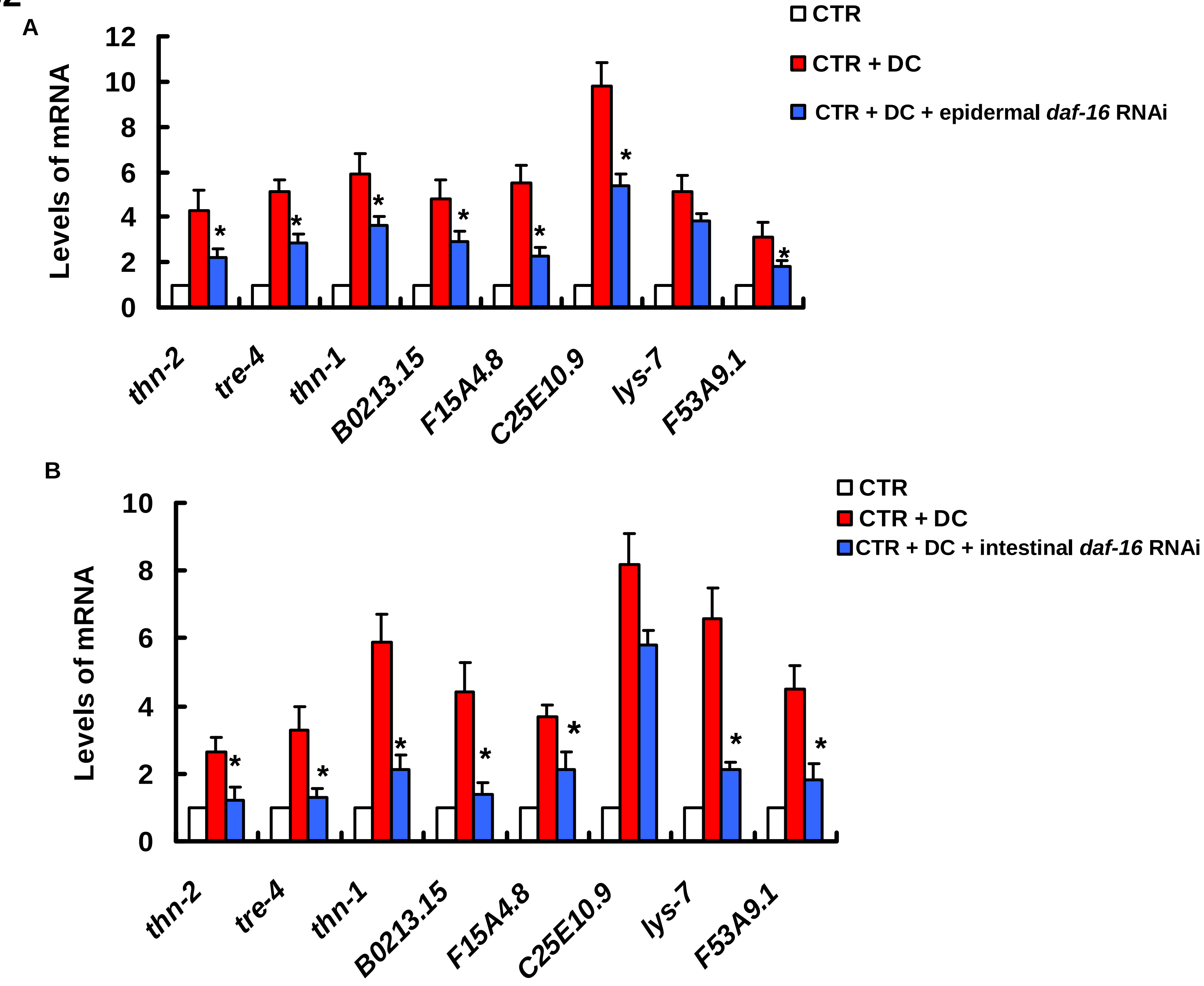
A
Levels of mRNA
12
10
8
6
4
2
0
CTR
CTR + DC
CTR + DC + epidermal daf-16 RNAi
thn-2
tre-4
thn-1
B0213.15
F15A4.8
C25E10.9
lys-7
F53A9.1
B
Levels of mRNA
10
8
6
4
2
0
CTR
CTR + DC
CTR + DC + intestinal daf-16 RNAi
thn-2
tre-4
thn-1
B0213.15
F15A4.8
C25E10.9
lys-7
F53A9.1

Figure S3

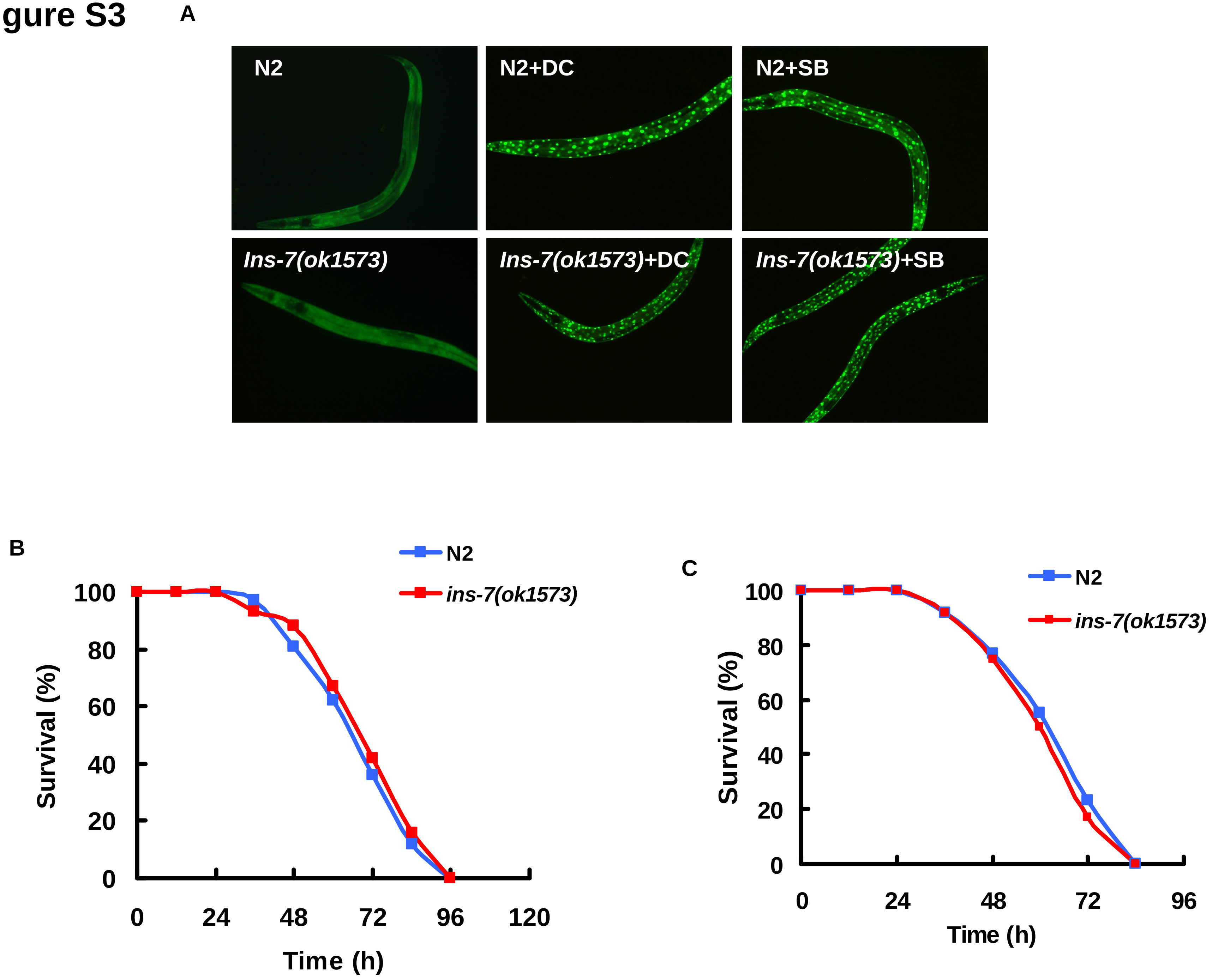
A
N2
N2+DC
N2+SB
Ins-7(ok1573)
Ins-7(ok1573)+DC
Ins-7(ok1573)+SB
B
N2
ins-7(ok1573)
Survival (%)
100
80
60
40
20
0
0
24
48
72
96
120
Time (h)
C
N2
ins-7(ok1573)
Survival (%)
100
80
60
40
20
0
0
24
48
72
96
Time (h)

Figure S4

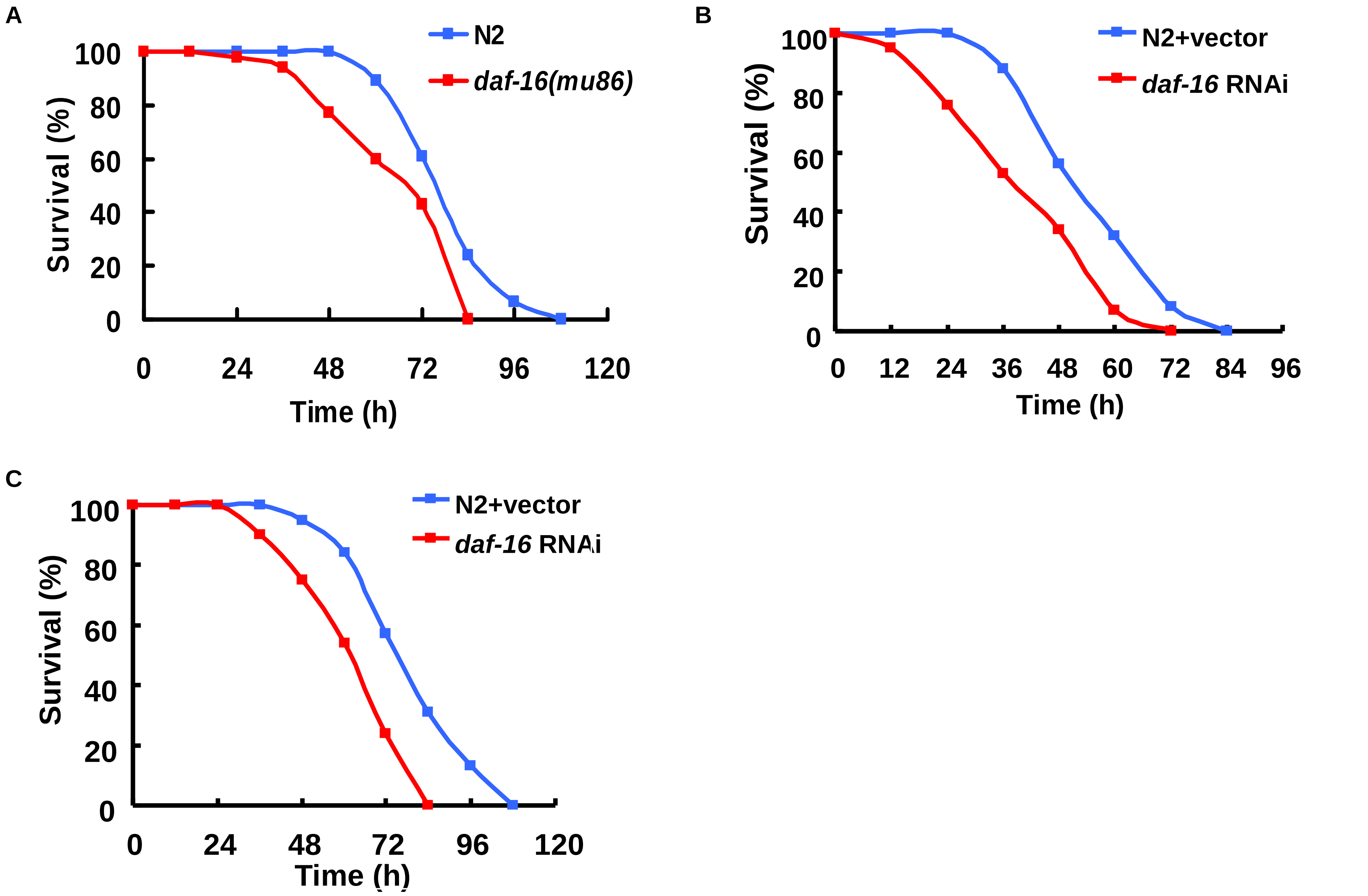
A
N2
daf-16(mu86)
Survival (%)
100
80
60
40
20
0
0
24
48
72
96
120
Time (h)
B
N2+vector
daf-16 RNAi
Survival (%)
100
80
60
40
20
0
0
12
24
36
48
60
72
84
96
Time (h)
C
N2+vector
daf-16 RNAi
Survival (%)
100
80
60
40
20
0
0
24
48
72
96
120
Time (h)

Figure S5

A B

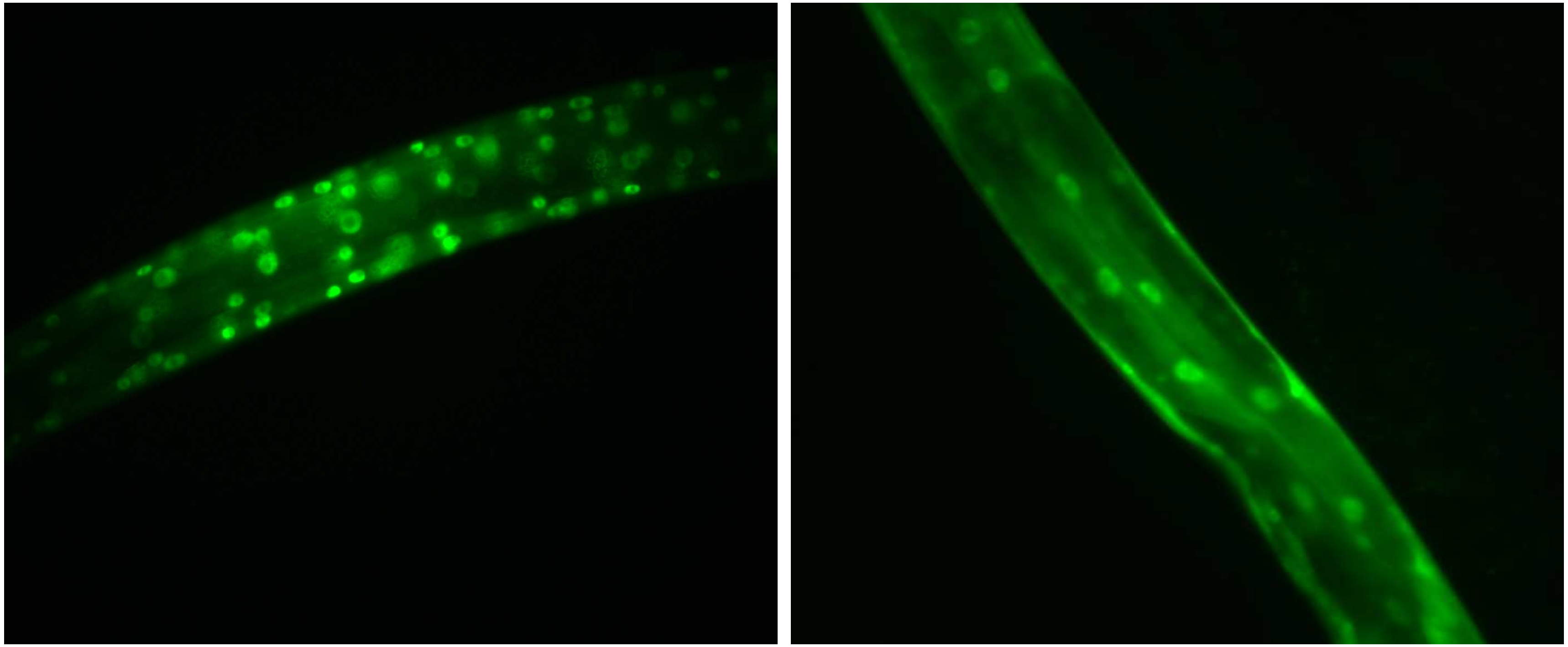

Figure S6

A

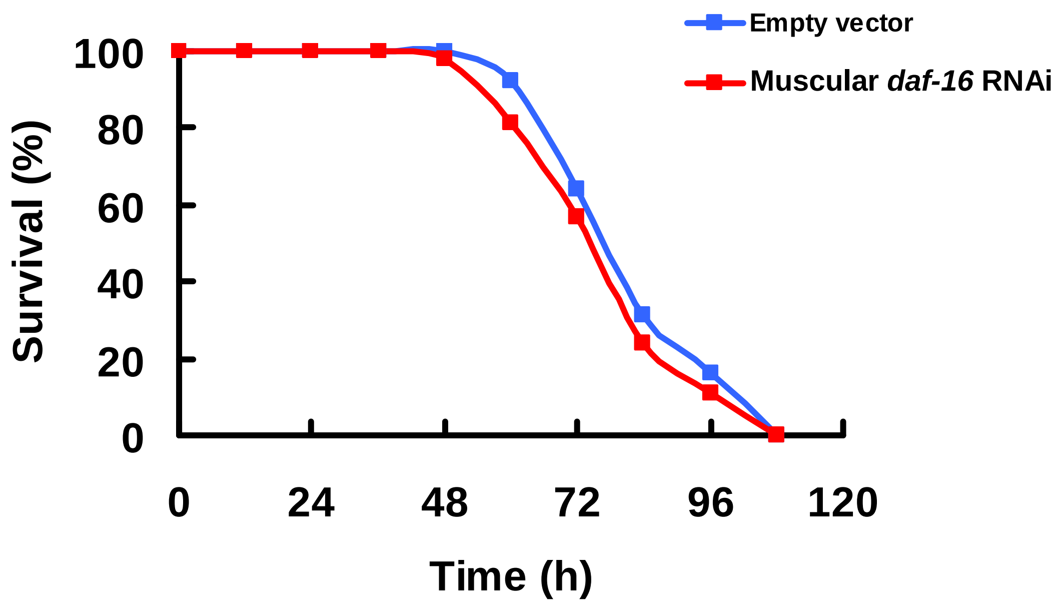
Empty vector
Muscular daf-16 RNAi
100
80
60
40
20
0
Survival (%)
0
24
48
72
96
120
Time (h)

B

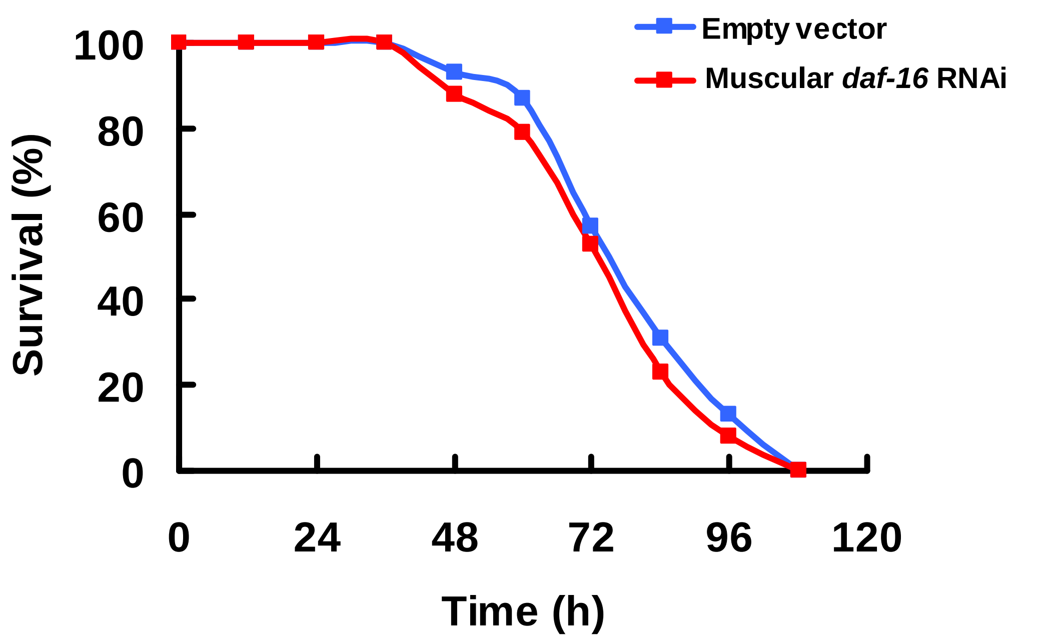
Empty vector
Muscular daf-16 RNAi
100
80
60
40
20
0
Survival (%)
0
24
48
72
96
120
Time (h)

Figure S7

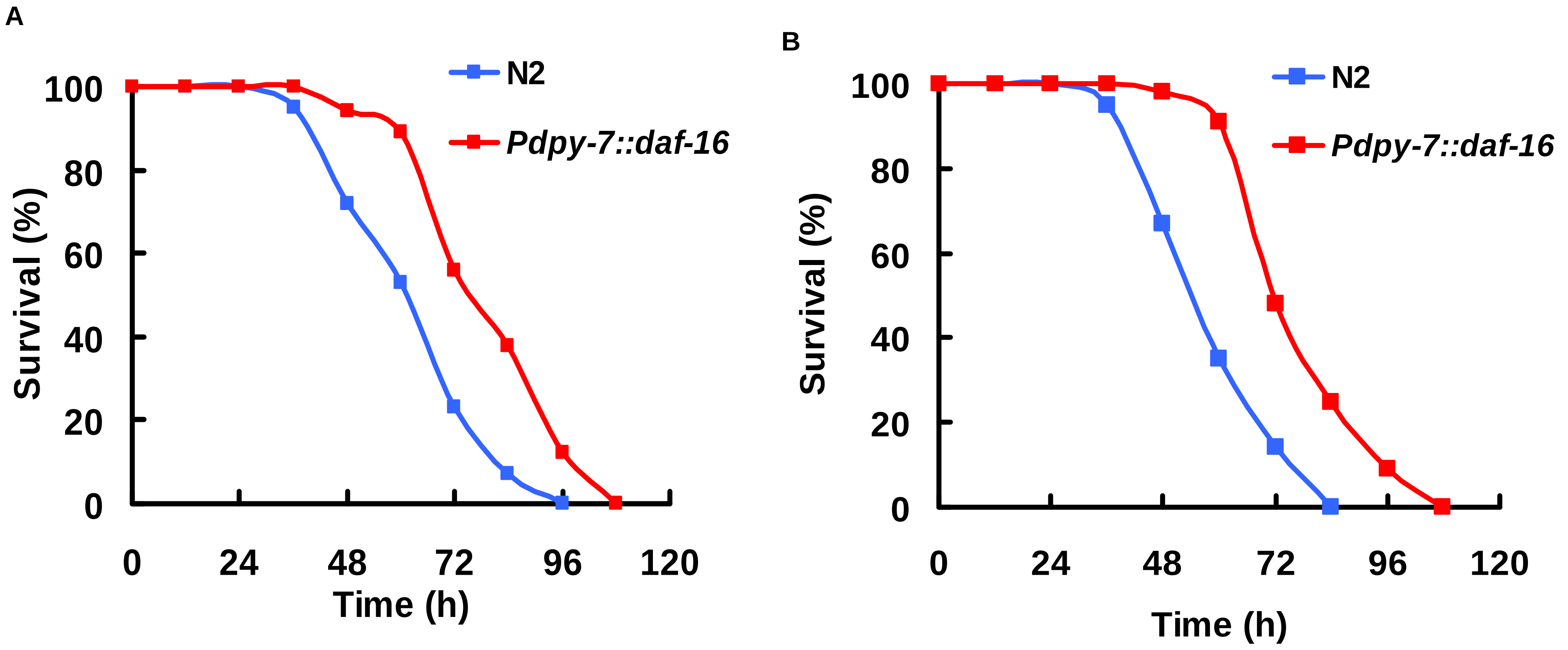

A
B
N2
Pdpy-7::daf-16
Survival (%)
Time (h)
0
20
40
60
80
100
0
24
48
72
96
120

Figure S8

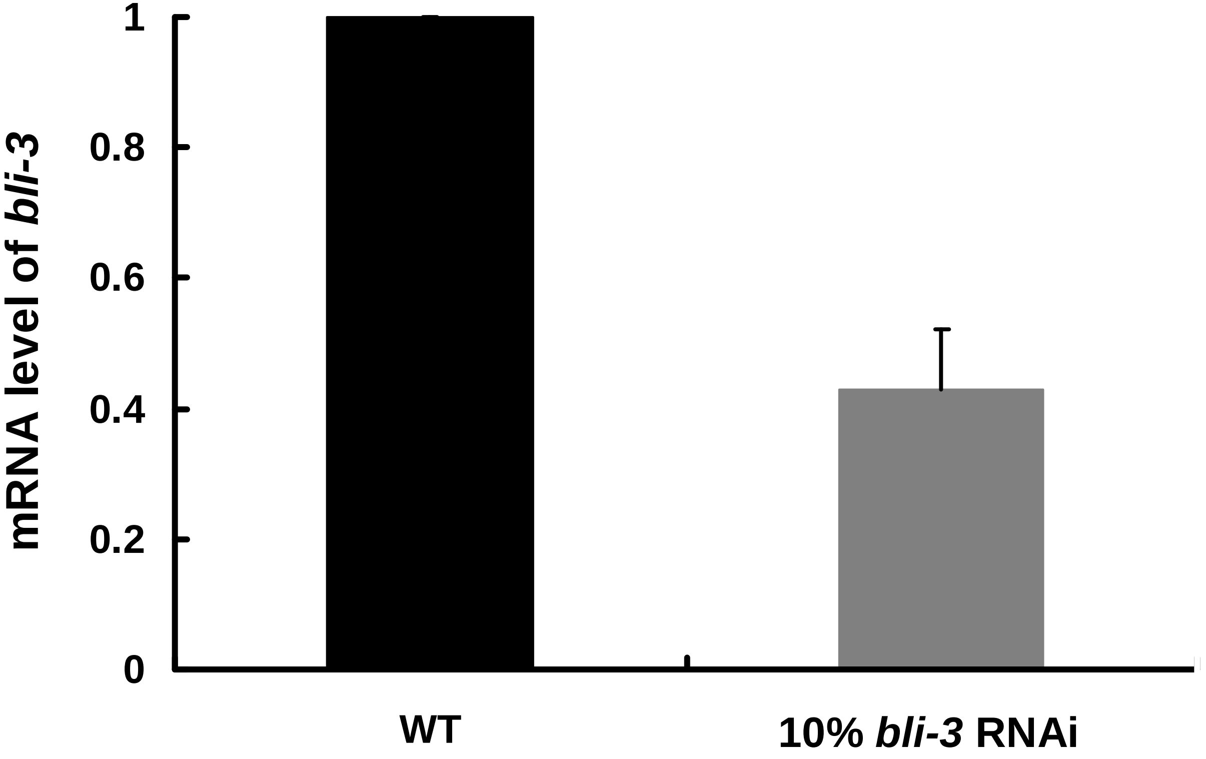

Figure S9

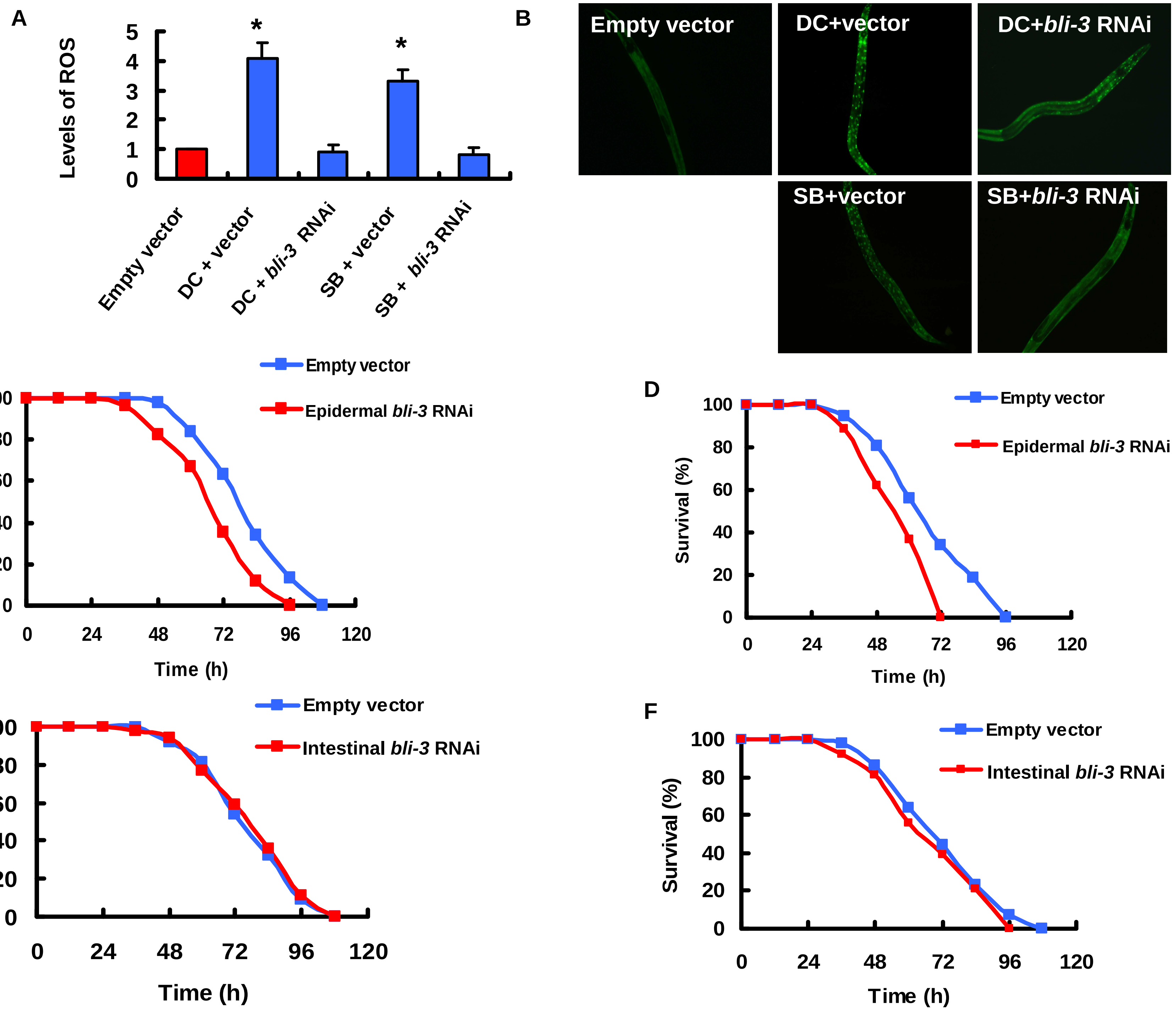
A
Levels of ROS
5
4
3
2
1
0
*
*
Empty vector
DC + vector
DC + bli-3 RNAi
SB + vector
SB + bli-3 RNAi
B
Empty vector
DC+vector
DC+bli-3 RNAi
SB+vector
SB+bli-3 RNAi
C
Empty vector
Epidermal bli-3 RNAi
Survival (%)
Time (h)
D
Empty vector
Epidermal bli-3 RNAi
Survival (%)
Time (h)
E
Empty vector
Intestinal bli-3 RNAi
Survival (%)
Time (h)
F
Empty vector
Intestinal bli-3 RNAi
Survival (%)
Time (h)

Figure S10

A

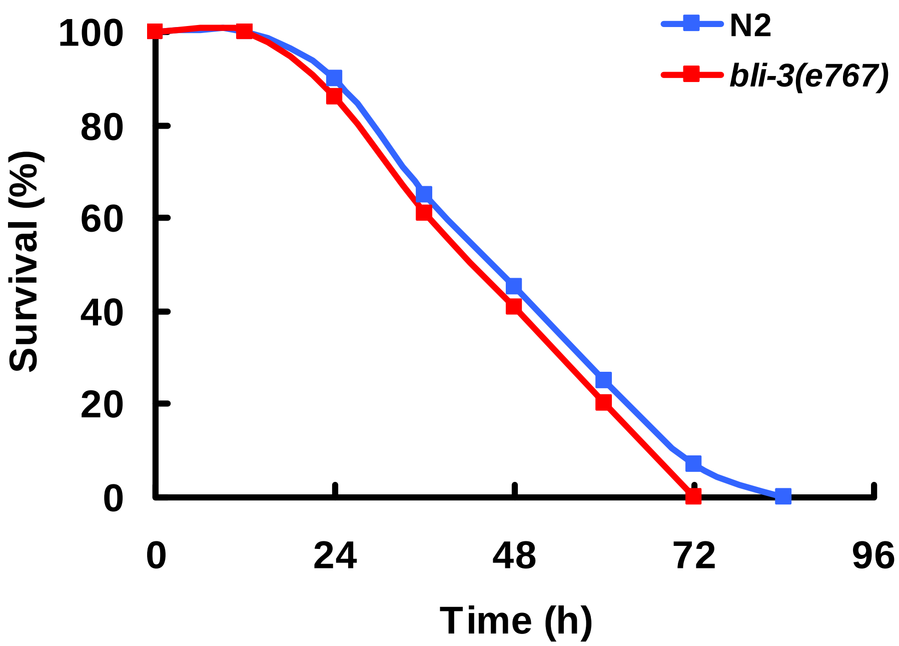

B

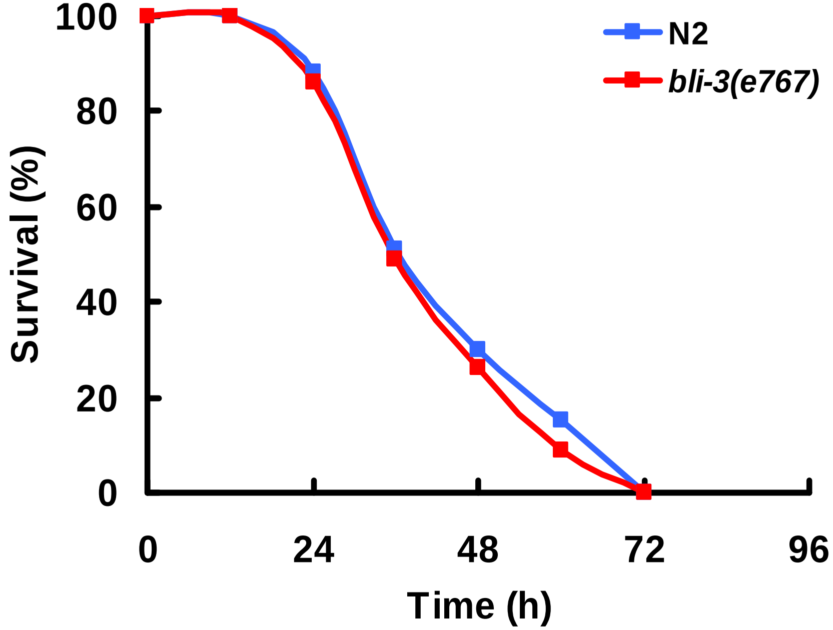

Figure S11

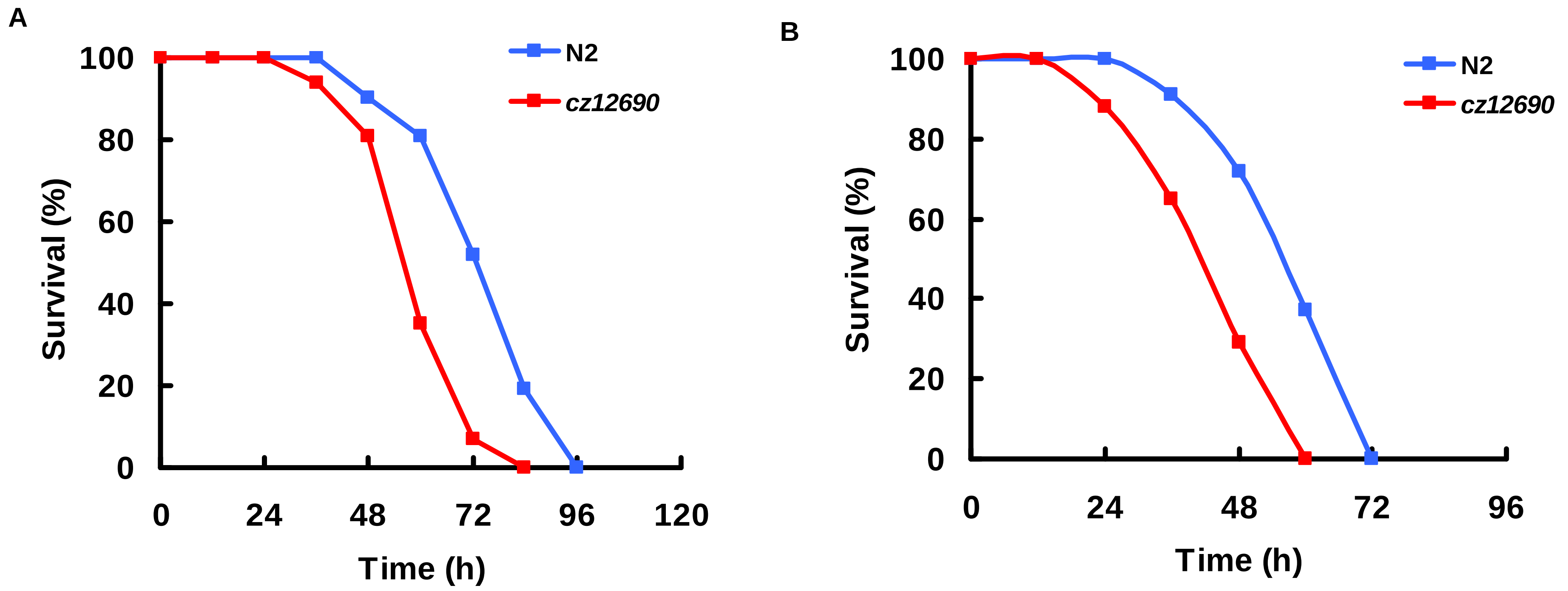
A
100
80
60
40
20
0
Survival (%)
0
24
48
72
96
120
Time (h)
N2
cz12690
B
100
80
60
40
20
0
Survival (%)
0
24
48
72
96
Time (h)
N2
cz12690

Figure S12

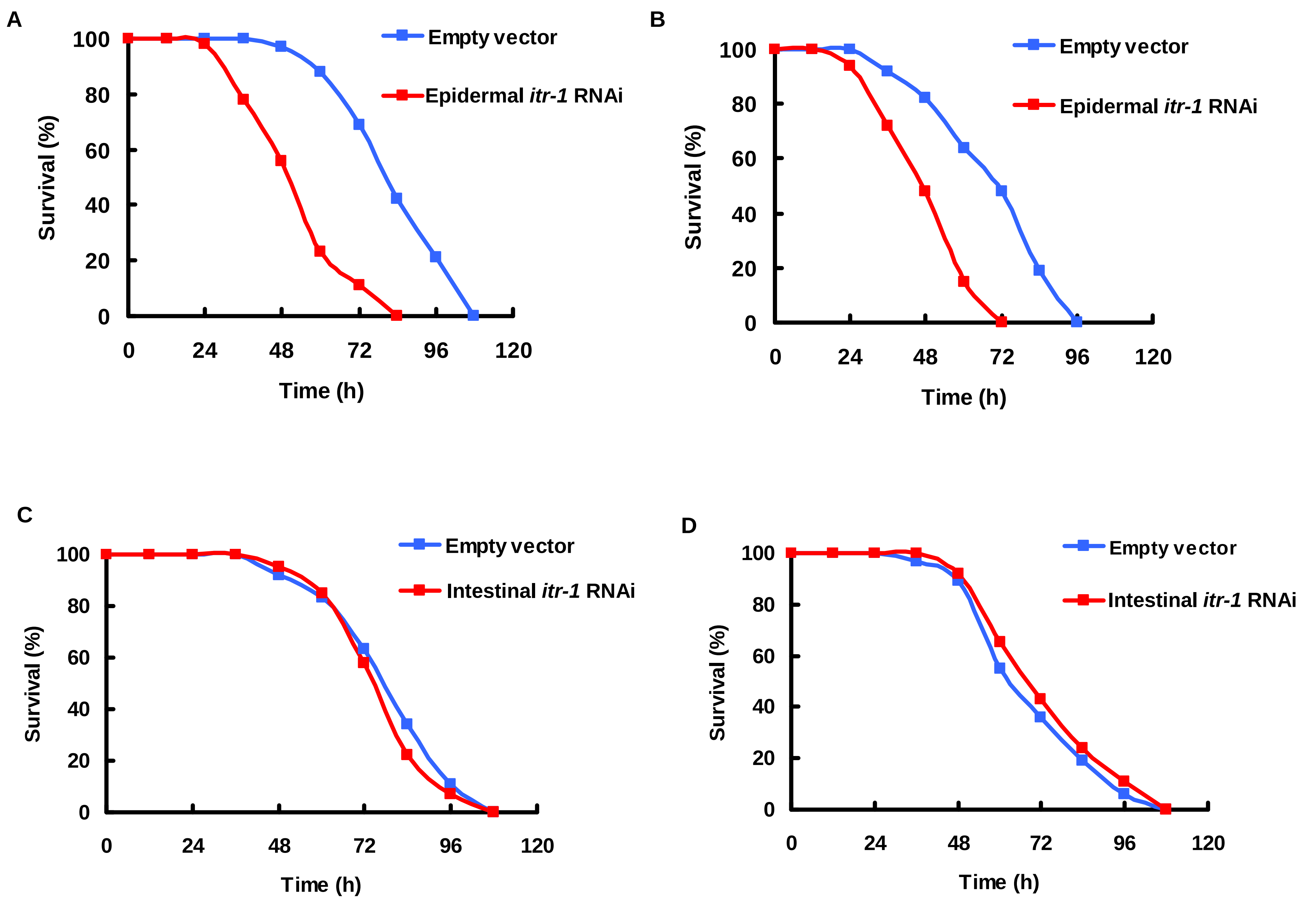
A
Empty vector
Epidermal itr-1 RNAi
Survival (%)
100
80
60
40
20
0
0
24
48
72
96
120
Time (h)
B
Empty vector
Epidermal itr-1 RNAi
Survival (%)
Time (h)
C
Empty vector
Intestinal itr-1 RNAi
Survival (%)
Time (h)
D
Empty vector
Intestinal itr-1 RNAi
Survival (%)
Time (h)

Figure S13

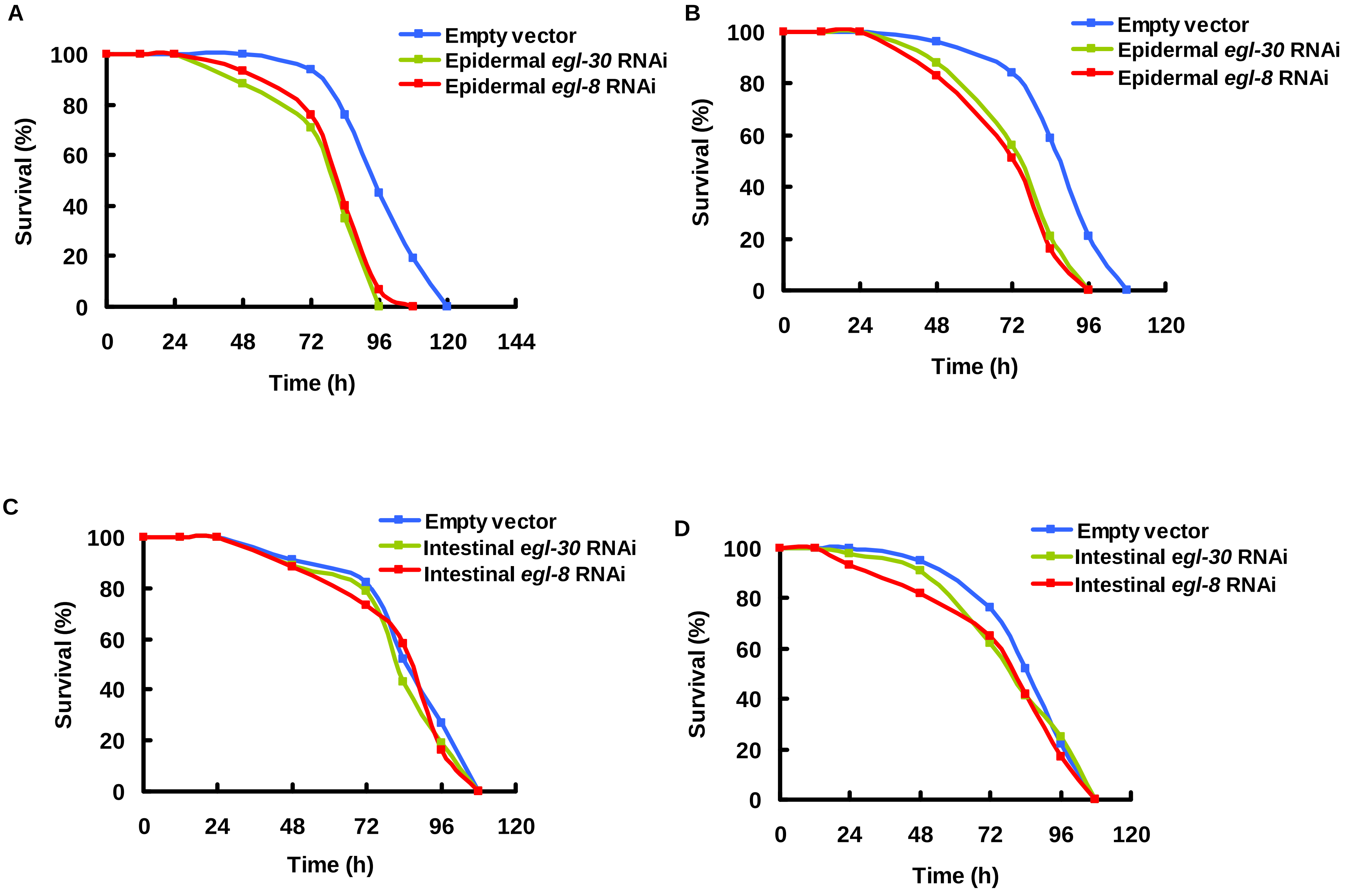
A
Empty vector
Epidermal egl-30 RNAi
Epidermal egl-8 RNAi
Survival (%)
100
80
60
40
20
0
0
24
48
72
96
120
144
Time (h)
B
Empty vector
Epidermal egl-30 RNAi
Epidermal egl-8 RNAi
Survival (%)
100
80
60
40
20
0
0
24
48
72
96
120
Time (h)
C
Empty vector
Intestinal egl-30 RNAi
Intestinal egl-8 RNAi
Survival (%)
100
80
60
40
20
0
0
24
48
72
96
120
Time (h)
D
Empty vector
Intestinal egl-30 RNAi
Intestinal egl-8 RNAi
Survival (%)
100
80
60
40
20
0
0
24
48
72
96
120
Time (h)

Figure S14

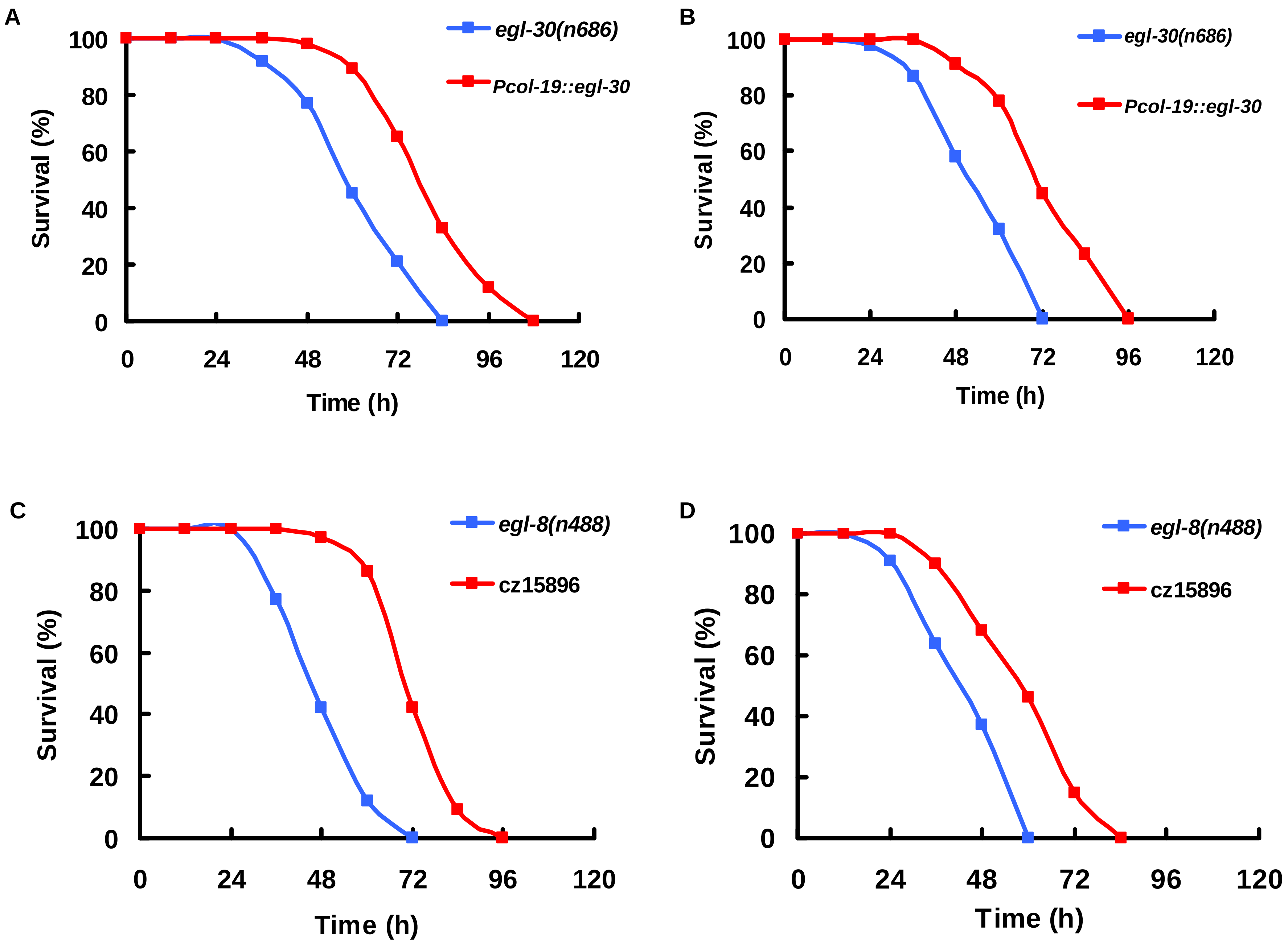
A
Survival (%)
Time (h)
egl-30(n686)
Pcol-19::egl-30
B
Survival (%)
Time (h)
egl-30(n686)
Pcol-19::egl-30
C
Survival (%)
Time (h)
egl-8(n488)
cz15896
D
Survival (%)
Time (h)
egl-8(n488)
cz15896

Figure S15

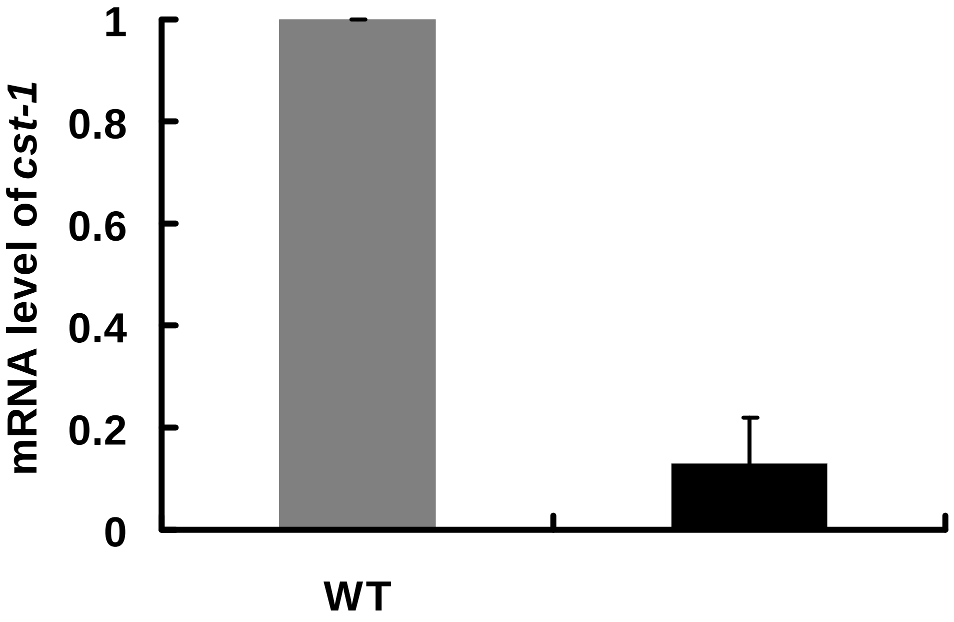

Figure S16

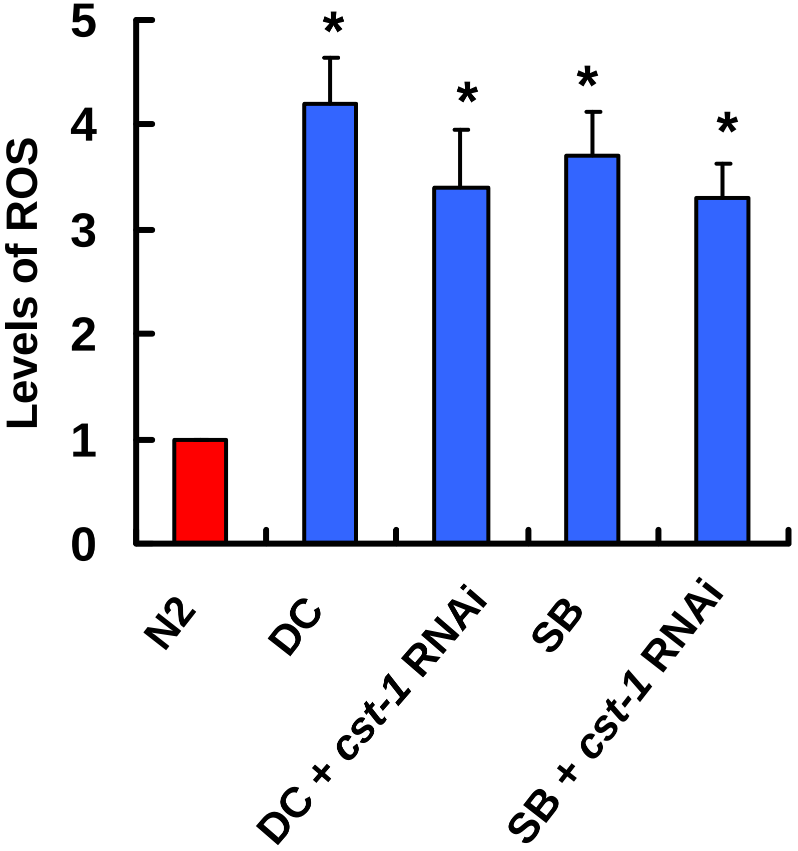

Figure S17

A

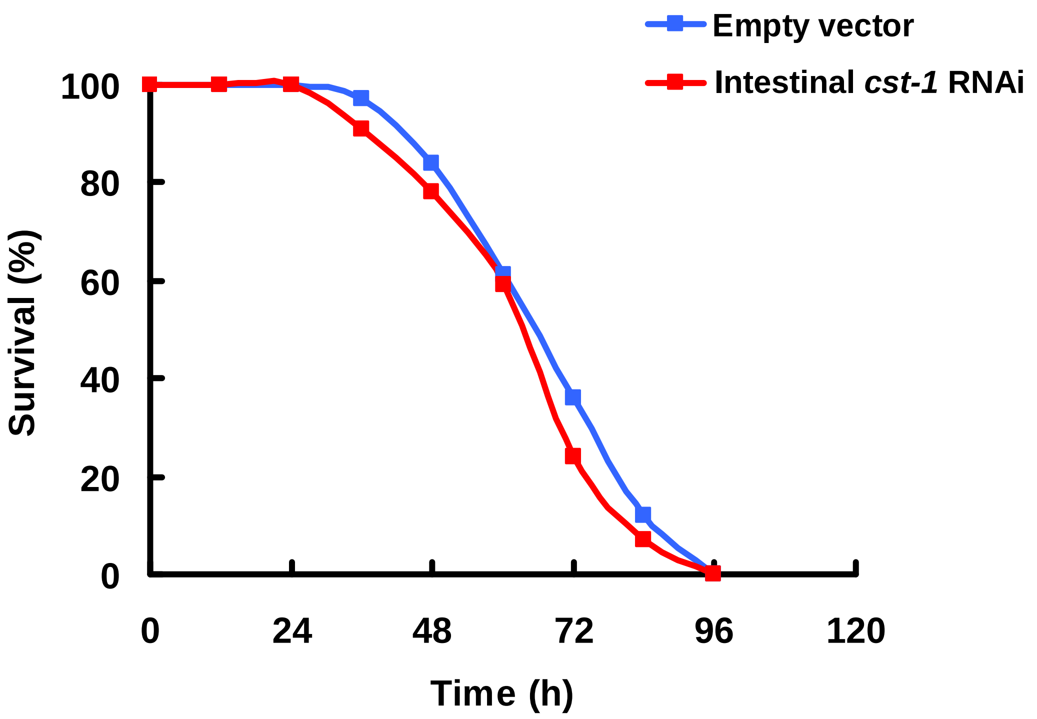

B

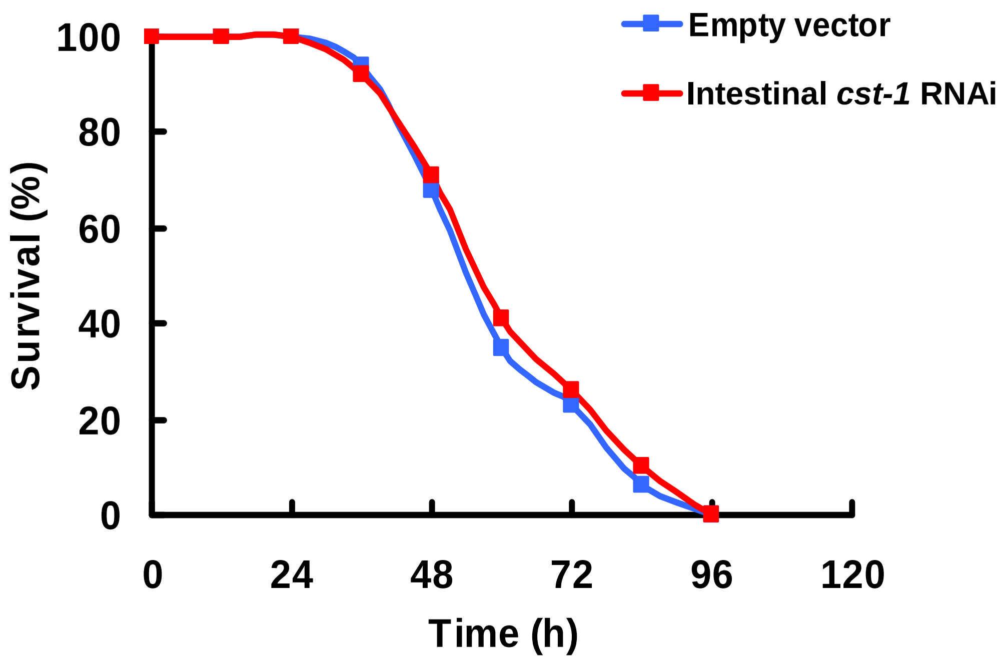

Figure S18

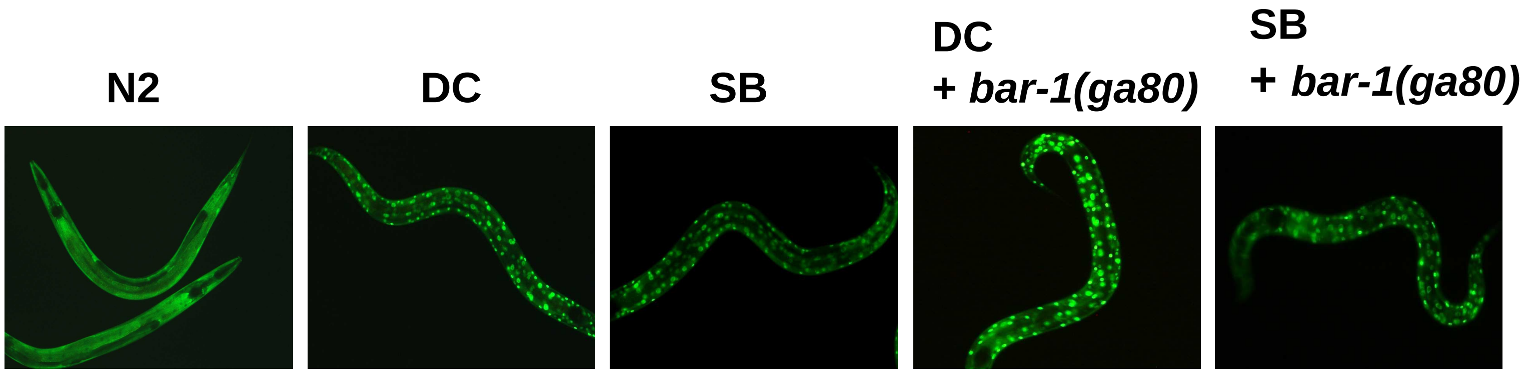

Supplement: Text S1 — Supporting Figures. Figure S1. Fungal infection and mutation in daf-12 induce DAF-16 nuclear translocation. (A) Wild-type worms in the absence of D. coniospora. (B) Wild-type worms were exposed to D. coniospora for 12 h. (C) daf-2 mutants were growth under normal conditions without D. coniospora. Figure S2. Both epidermal- and intestinal-specific knock-down of daf-16 by RNAi suppress the expression of DAF-16 target genes. (A) qPCR analysis of expression of DAF-16 target genes in NR222 strains (CTR), CTR 24 h after D. coniospora infection (CTR+DC), and CTR subjected to daf-16 RNAi after D. coniospora infection (CTR+DC+epidermal daf-16 RNAi). (B) qPCR analysis of expression of the intestinal-specific RNAi strain sid-1(qt9);Is[sur-5::GFP]; alxIs7[VHA-6p::SID-1::SL2::GFP] (CTR), CTR 24 h after D. coniospora infection (CTR+DC), and CTR subjected to daf-16 RNAi after D. coniospora infection (CTR+DC+epidermal daf-16 RNAi). *P<0.05, CTR+DC relative to CTR+DC+ daf-16 RNAi. Figure S3. Genetic loss of ins-7 has no effect on DAF-16 translocation and the survival of worms after D. coniospora infection and physical injury. (A) Mutation in ins-7(ok1573) did not influence the nuclear accumulation of DAF-16 after D. coniospora infection (DC) and treatment with spiny balls (SB). (B and C) Mutation in ins-7(ok1573) did not affect the survival of nematodes after D. coniospora infection (B) and treatment with spiny balls (C). Figure S4. DAF-16 is required for resistance to fungal infection. (A) daf-16(mu86) mutants were sensitive to C. rosea infection. (B–C) daf-16 RNAi reduced the survival rate of nematodes exposed to D. coniospora (B) and C. rosea (C). P<0.001 relative to wild-type animals. Figure S5. Epidermal-specific knock-down of daf-16 reduces the expression of daf-16 in the hypodermis. (A) NR222 strains were exposed to D. coniospora for 12 h. (B) NR222 strains subjected to daf-16 RNAi were exposed to D. coniospora for 12 h. Figure S6. Muscular-specific daf-16 RNAi has no effect [file ppat.1003660.s003.pdf]
